# Simultaneous inhibition of ID1 and ID3 mitigates fibroblast activation via cell cycle and MEK/ERK pathways in pulmonary fibrosis

**DOI:** 10.7150/thno.127118

**Published:** 2026-04-16

**Authors:** Samar A Antar, Eric Mensah, Jacob Dahlka, Mark C. Renton, Ahmed A Raslan, Michael Aziz, Aymen Halouani, Seun Imani, Aanandi Parashar, Scott R. Johnstone, Robert Benezra, Diego Fraidenraich, Giovanni Ligresti, Yassine Sassi

**Affiliations:** 1 Fralin Biomedical Research Institute at Virginia Tech Carilion, Virginia, USA.; 2 Department of Pharmacology and Biochemistry, Faculty of Pharmacy, Horus University-Egypt, New Damietta 34518, Egypt.; 3 Icahn School of Medicine at Mount Sinai, New York, USA.; 4 Arthritis and Autoimmune Diseases Center, Department of Medicine, Boston University Chobanian and Avedisian School of Medicine, Boston, MA, USA.; 5 Pulmonary Center, Department of Medicine, Boston University Chobanian and Avedisian School of Medicine, Boston, MA, USA.; 6 Department of Zoology, Faculty of Science, Assiut University, Assiut, Egypt.; 7 Cancer Biology and Genetics Program, Sloan Kettering Institute, Memorial Sloan Kettering Cancer Center, New York, NY, USA.; 8 Department of Cell Biology & Molecular Medicine, Rutgers New Jersey Medical School, Newark, New Jersey, USA.; 9 Department of Biomedical Sciences and Pathobiology, Virginia-Maryland College of Veterinary Medicine, Virginia Tech, Blacksburg, Virginia, USA.; 10 Department of Internal Medicine, VTC School of Medicine, Roanoke, Virginia, USA.

**Keywords:** ID proteins, idiopathic pulmonary fibrosis, lung fibroblast, bleomycin

## Abstract

**Background:**

Idiopathic pulmonary fibrosis (IPF) is a fatal lung disease for which novel therapeutic approaches are urgently needed. Transforming Growth Factor-β (TGF-β) plays a central role in IPF pathogenesis by activating lung fibroblasts. Inhibitor of DNA binding (ID) proteins are regulated by TGF-β; however, their role in IPF remains poorly understood. We aimed to evaluate the regulation of ID proteins in IPF and to determine their functional role in human lung fibroblasts (HLF) *in vitro* and pulmonary fibrosis *in vivo*.

**Methods:**

ID protein expression was assessed in lungs and lung fibroblasts from mice and patients with pulmonary fibrosis. *In vitro*, the effects of ID1/ID3 inhibition and overexpression on HLF proliferation, migration and differentiation into myofibroblasts were evaluated. Genetic and pharmacological approaches were used *in vivo* to determine the role of ID1/ID3 in pulmonary fibrosis.

**Results:**

ID1/ID3 levels were elevated in lungs and lung fibroblasts from mice and patients with pulmonary fibrosis, as well as in HLFs treated with TGF-β. ID1/ID3 knockdown reduced proliferation, migration and differentiation of healthy and IPF-derived HLFs. Bleomycin-exposed ID1/ID3 double KO mice exhibited improved lung function and reduced fibrosis compared with WT mice. Pharmacological inhibition of ID1/ID3 decreased HLF proliferation, migration and differentiation *in vitro* and attenuated pulmonary fibrosis *in vivo*. Lung-specific inhibition of ID1/ID3 using adeno-associated viruses expressing short hairpins targeting ID1 and ID3 also reversed pulmonary fibrosis in mice. Mechanistically, ID1/ID3 inhibition reduced fibroblast proliferation through regulation of cell cycle genes and attenuated fibroblast differentiation via the MEK/ERK pathway.

**Conclusions:**

Simultaneous inhibition of ID1 and ID3 attenuates pulmonary fibrosis. Targeting ID1/ID3 represents a potential novel therapeutic strategy for IPF.

## Introduction

Idiopathic pulmonary fibrosis (IPF) is an irreversible, chronic, progressive fibrosing lung disease of unknown cause with a median survival time of 3-5 years post-diagnosis 1, 2. IPF affects older adults and is characterized by a gradual decline in lung function and worsening dyspnea, resulting in a poor prognosis 3-6. The exact causes and mechanisms of IPF remain incompletely understood; however, the disease is characterized by excessive deposition of extracellular matrix (ECM), largely driven by the activation of fibroblasts 7-9. Under normal conditions, fibroblasts remain in a quiescent state; however, in response to tissue injury, they become activated and differentiate into myofibroblasts, which are responsible for ECM production and tissue remodeling 10-12. Cytokines, particularly transforming growth factor-β (TGF-β), have been identified as key mediators of fibroblast activation^13^. As currently approved therapies only slow disease progression, a substantial unmet medical need remains in the treatment of IPF 14, 15. A primary therapeutic goal is to reduce tissue fibrosis, which may be achieved by enhancing pathways that counteract fibrotic processes and/or inhibiting profibrotic signals and mediators.

Inhibitors of DNA Binding (ID1-4) proteins are a subgroup of the helix-loop-helix (HLH) transcription factor family, that play a crucial role in regulating cell fate 16-18. However, unlike other HLH members, they lack a DNA-binding motif 17, 19. ID proteins function as dominant negative regulators of other HLH proteins by inhibiting their DNA binding and transcriptional activity 17. These proteins are critical during development, where they regulate cell-cycle progression, cell proliferation, differentiation, cell fate determination, hematopoiesis, angiogenesis, and the metabolic adaptation of cancer cells 20-23. ID gene expression is regulated by a wide range of growth factor and cytokine signaling cascades including TGF-β 24. However, the role of ID proteins in IPF pathogenesis remain poorly understood. Addressing these knowledge gaps by elucidating the role of ID proteins in IPF pathogenesis and progression could reveal novel therapeutic avenues, directly responding to the urgent need for more effective IPF treatments.

Building on these observations, the present study aimed to investigate the regulation and role of ID proteins in pulmonary fibrosis using a combination of genetic and pharmacological approaches. We used human lung fibroblasts (HLF) isolated from the lungs of healthy donors and patients with IPF to assess ID protein expression and investigate their functional effects *in vitro*. Our data show that ID1 and ID3 levels are upregulated in lung fibroblasts from mice and humans with IPF, and in lung fibroblasts treated with TGF-β. We subsequently used a pharmacological inhibitor of ID1/ID3 and generated ID1/ID3 double KO mice to determine the role of ID1 and ID3 in pulmonary fibrosis. Our study demonstrates that simultaneous inhibition of ID1 and ID3 attenuates the proliferation, migration, and differentiation of human lung fibroblasts* in vitro*, and pulmonary fibrosis *in vivo* in mice.

## Materials and Methods

### Human lung samples

Lung tissues from patients with IPF were obtained from explanted lungs at the time of transplantation. All patients provided written informed consent, and the study was approved by the University of Michigan Institutional Review Board (Ann Arbor, MI; HUM00105694). The diagnosis of IPF was established based on clinical and pathological criteria. Normal control lung tissues were obtained from deceased donors whose lungs were deemed unsuitable for transplantation and were provided by Gift of Life Michigan with family consent for research use. No compensation was provided to patients or donor families for tissue procurement. Sex and/or gender were not considered as variables in the study design. Both sexes were included and randomly distributed across experimental groups, and no sex-dependent differences were observed. Due to the limited sample size, statistical analyses stratified by sex or gender were not performed. The patients' characteristics are shown in Table S1.

### Human lung fibroblasts

Normal (healthy) human lung fibroblasts (Lonza; cat #CC-2512) isolated from 6 healthy donors, and IPF-diseased human lung fibroblasts (Lonza; cat#CC-7231) isolated from 6 patients with IPF were purchased from Lonza and cultured according to the recommended guidelines. The cells were passed upon reaching confluency and were used at passages 2 to 7. Primary human lung fibroblasts were cultured in fibroblast growth medium (FGM; Lonza, Cat# CC-3132) consisting of basal medium supplemented with fetal bovine serum (FBS), insulin, human fibroblast growth factor (hFGF), gentamicin/amphotericin-B, and additional fibroblast growth supplements, according to the manufacturer's instructions. Cells were maintained at 37 °C in a humidified incubator with 5% CO₂ and passaged at 80-90% confluence before being treated with adenoviral vectors at a multiplicity of infection (MOI) of 30. The vectors included Ad-LacZ, Ad-ID1, Ad-ID3, Ad-Cdk1, Ad-Egr1, and Ad-MEK1, all sourced from Vector Biolabs. Following a 48h incubation, Adenovirus-treated cells were then harvested for RNA and protein extraction. All cells used in this study were tested by Lonza to confirm the absence of HIV-1, HBV, HCV, mycoplasma, bacteria, yeast and fungi.

### Reagents

AGX51 (MedChem Express) was used at a final concentration of 20 μM. For the *in vivo* studies, AGX51 was administered intraperitoneally to mice at a dose of 50 mg/kg, twice daily, three days per week. AAV1-GFP-U6-mID1-shRNA (shAAV-261835), AAV1-GFP-U6-mID3-shRNA (shAAV-261838), Ad-h-ID1 (ADV211761), Ad-h-ID3 (ADV-211764), Ad-MEK1 (ADV-214913), Ad-Egr1 (ADV-207671), and Ad-Cdk1 (1764) were purchased from Vector Biosystem. Recombinant human Transforming growth factor-β1 (TGF-β1, Peprotech) aliquoted and kept at -20 °C until use. TGF-β1 was used at concentration of 5 ng/ml. MEK1 inhibitor (Selumetinib, 10µM, MedChem Express) was aliquoted and kept at -80 °C until use.

### AAV delivery

Mice were anesthetized by IP injection of xylazine and ketamine and were secured to a tray in the supine position. Subsequently, using a 20 G angiocath, animals were intubated. The board was tilted at 45 degrees and the IA-1C Micro sprayer tip (PennCentury) was inserted through the lumen of the angiocath. AAV1-shRNA-ID1/ID3 (Vector Biolabs) and AAV1-Luciferase (AAV-Ctrl) were administered via IT delivery (3x10^11^ genome copies per mouse). The animals were then extubated and returned to their cages.

### Generation and use of ID1/ID3 knock-out mice

ID1/ID3 double knock-out mice were generated by crossing mice carrying a ubiquitous deletion of ID3 with mice carrying a fibroblast specific deletion of ID1 (by crossing ID1^fl/fl^ mice with Col1a2-CreER mice (JAX stock #029567)). Genotyping of these mice was performed by PCR. Mice were administered daily intraperitoneal (i.p.) injections of tamoxifen (TAM, 40 mg/Kg; Sigma) for five consecutive days. All animal procedures were conducted according to the Institutional Animal Experimentation Guidelines. The mice were housed in a pathogen-free facility, and all animal experiments were approved by the Animal Care and Use Committees of Virginia Tech.

### Quantitative RT-PCR

Real-time qPCR was conducted using the PerfeCTa SYBR^TM^ Green PCR Fast Mix Kit (VWR) following the manufacturer's protocol. A melting curve analysis was performed to verify primer specificity. Gene expression levels were normalized to the housekeeping gene GAPDH, and relative expression was calculated using the ΔΔCt method by comparing Ct values of the experimental groups to controls. The primers sequences are listed in Table S2.

### Bulk RNA sequencing and data analysis

HLFs were seeded in 6-well plates at a density of 1 × 10⁵ cells per well. The following day, cells were treated with media containing 0.1% FBS, 5%FBS, or 5%FBS+AGX51 (20 µM) for 48 h. Cells were then washed twice with ice-cold PBS, and total RNA was extracted using TRIzol reagent (ThermoFisher) according to the manufacturer's instructions. Briefly, cells were homogenized in TRIzol, and following phase separation, the aqueous phase containing RNA was collected. RNA was precipitated with isopropanol, washed with 75% ethanol, and resuspended in RNase-free water. Samples were treated with DNase I (Thermo Scientific) to remove genomic DNA contamination. RNA concentration and purity were determined using a NanoDrop One spectrophotometer (ThermoFisher), and samples were stored at -80 °C until further processing.

RNA integrity analysis, library preparation, sequencing, and raw read count generation were performed commercially by Novogene (Sacremento, CA, USA) using their Human mRNA Sequencing pipeline. Full parameters, protocols, and quality control reports are available on request, but are explained briefly below:

RNA integrity was assessed using the 5400 Fragment Analyzer System (Agilent Technologies, CA, USA). mRNA library preparation was performed with poly A enrichment and library quality was verified using Qubit assay for quantification, Agilent Bioanalyzer for size distribution, and qPCR for molarity using adaptor-specific primers. Sequencing was performed on the NovaSeq X Plus (Illumina, USA) platform with 150 bp paired-end reads and a minimum of 6 G raw data. Raw sequencing data in FASTQ format were cleaned using fastp. Reads were then aligned to the human genome (hg38) using 'HISAT2' (v2.0.5). Raw read counts were generated using 'featureCounts' (v1.5.0). Raw count normalization was performed using 'DESeq2' (v1.46.0) 25 in the R statistical analysis program (v4.4.3). To exclude low count genes prior to 'DESeq2' normalization, only genes with at least 10 counts in one sample were kept. Gene symbols were extracted based on provided Ensembl ID using 'clusterProfiler' (v4.14.6) 26 and 'org.Hs.eg.db' (v3.20.0). 'DESeq2' normalized counts were log_2_ transformed to ensure a normal distribution, and euclidean distances were calculated for principal component analysis (PCA).

Differential gene expression analysis was performed using 'DESeq2' with Benjamini-Hochberg multiple comparison adjustment and 'apeglm' (v1.28.0)^27^ fold change (FC) shrinkage. An adjusted p-value (padj) < 0.05 and an |log_2_(FC)| > 0 was set as the threshold for differentially expressed genes (DEGs). Applying a |log2FC| > 0.1 threshold instead of |log2FC| > 0 would have a negligible impact on the results, excluding only one gene per comparison. Volcano plots displaying log_10_(padj) and log_2_(FC) of DEGs were generated in R.

Pathway enrichment analysis was performed on DEGs with a padj < 0.05 with 'clusterProfiler' in R using the Gene Ontology (GO) database. The list of detected genes in all samples after low gene count exclusion was used as the background gene set. The GO terms with a padj < 0.05 following Benjamini-Hochberg multiple comparison adjustment were considered statistically significantly enriched. Separate pathway enrichment analysis was performed for upregulated DEGs (log_2_(FC) > 0) and downregulated DEGs (log_2_(FC) < 0).

Transcription factor inference was estimated using the univariate linear model method in 'decoupleR' (v2.12.0) 28 'DESeq2' gene-level Wald statistics were used as the gene input and 'DoRothEA' (v1.18.0) 29 regulatory network was used as the transcription factor-gene target database. Only transcription factors with at least five target genes present in the dataset were considered. P-values were then corrected using Benjamini-Hochberg multiple comparison adjustment.

### Single-cell RNA sequencing and data analysis

Processed feature and barcode matrices from a publicly available human scRNA-seq dataset from Tsukui *et al*. (GSE132771) 30 were imported and analyzed using BioTuring BrowserX Pro (https://academic.bioturing.com/bbrowserx/). The reanalysis of GSE132771 was performed using the same methods and strategy as described in the original publication. Unsupervised dimensionality reduction was performed using Uniform Manifold Approximation and Projection (UMAP) with canonical correlation analysis (CCA) subspace alignment, followed by unsupervised graph-based clustering. Analysis of representative marker genes was used to identify clusters corresponding to endothelial, epithelial, mesenchymal, and hematopoietic cell populations. The indicated cell populations were then selected and further re-clustered for downstream analyses. Cell-type annotations were based on those reported by Tsukui *et al*. Both normal and IPF cells were included in the analysis. Differentially expressed genes were identified using the following criteria: log2 fold change ≤ -0.1 or ≥ 0.1 and Benjamini-Hochberg adjusted P value ≤ 0.05.

### Immunoblot analysis

To analyze the protein content, cell lysates were prepared using RIPA lysis buffer (Sigma-Aldrich) supplemented with a protease and a phosphatase inhibitor cocktail (Sigma-Aldrich). After centrifugation at 12,000 × g for 20 min at 4 °C, supernatants were collected, and protein concentrations were determined using the BCA Protein Assay Kit (Pierce, ThermoFisher). Samples were stored at -80 °C until analysis. The proteins were then separated using SDS-polyacrylamide gel electrophoresis (PAGE) and transferred onto nitrocellulose membranes. The membranes were blocked with 5% bovine serum albumin (BSA) for 1 hour. The membranes were then incubated overnight with the primary antibodies (1:1000 dilution) listed in Table S3. After washing three times for 10 minutes in Tris-buffered saline with 0.1% Tween 20 (TBST; Boston BioProducts), the membranes were incubated with appropriate horseradish peroxidase (HRP)-conjugated secondary antibodies (1:2000 dilution, Cell Signaling) for 1h at RT. After three further washes, Protein bands were detected using the Bio-Rad ChemiDoc Touch Imaging System with SuperSignal™ West Pico PLUS Chemiluminescent Substrate (Thermo Scientific). Densitometric analysis of the bands was performed using Image Lab software (Bio-Rad), and relative protein expression was normalized to control protein levels for each sample.

### Lung function

Anesthetized mice were intubated with a sterile blunt 19G cannula, which was then connected to a flexiVent respirator (SCIREQ). The 19G catheters were selected because they did not cause any injury to the trachea during intubation. Prior to each experiment, the flexiVent system was calibrated to account for the mechanical properties of both the system and the tracheal cannula, ensuring accurate calculations of lung mechanical properties. Ventilation was set with a tidal volume of 10 ml/kg body weight, a respiratory rate of 150 breaths per minute, and a positive end-expiratory pressure (PEEP) of 3 cm H_2_O. Two recruitment maneuvers (deep lung inflation at 30 cm H_2_O) and three pressure-controlled quasi-static pressure-volume (PV) loops were applied. The inspiratory capacity (IC), which indicates the amount of air that can be inhaled following a normal expiration, was calculated during deep inflation. This maneuver helps open closed lung areas, restore airway patency, and normalize lung volume. Tissue elastance, which reflects lung resistance to distension under mechanical load, was calculated by fitting the constant-phase model to impedance spectra obtained during FOT. Quasi-static compliance (Cst), which represents lung distensibility, was determined as the mean of the three PV loop values using the Salazar-Knowles equation.

### Immunostaining

Formalin-fixed paraffin-embedded (FFPE) human lungs were cut in serial sections (5 μm). Slides were subsequently deparaffinized in xylene and rehydrated through a graded ethanol series. Antigen retrieval was performed using Universal Epitope Retrieval Buffer (Electron Microscopy Sciences) for 40 min in a Retriever 2100 pressure cooker. After cooling, sections were blocked for 2 h in 1× PBS containing 0.1% Triton X-100 and 5% goat serum to reduce nonspecific binding. The sections were then incubated overnight at 4 °C with primary antibodies against ID1 (sc-133103, Santa Cruz), ID3 (2B11, Invitrogen), and fibronectin (15613-1-AP, Proteintech). Following primary antibody incubation, slides were washed and incubated with species-appropriate fluorophore-conjugated secondary antibodies (Cell Signaling). Nuclei were counterstained with DAPI, and slides were mounted using an antifade mounting medium (Thermofisher). Fluorescence images were acquired using a Keyence fluorescence microscope under identical acquisition settings for all samples to allow direct comparison between control and IPF groups.

### Statistical analyses

All quantitative data are reported as means ± SEM. Statistical analysis was performed with the Prism software package (GraphPad Version 11). Data distribution was assessed by D'Agostino-Pearson omnibus test, Shapiro-Wilk test, and Kolmogorov-Smirnov test for normality. Depending on the distribution of data, unpaired t-test or Mann-Whitney test was used to determine statistical significance. Differences among multiple means were assessed by one-way or two-way ANOVA followed by Bonferroni correction or followed by Holm-Sidak's test analysis as appropriate. Ashcroft scores were analyzed using the Kruskal-Wallis test followed by Dunn's multiple-comparison test. P-values <0.05 were considered significant (corresponding symbols in figures are *for P < 0.05, **for P < 0.01, and ***for P < 0.001).

Additional experimental materials and methods are provided in the **Supplementary Information.**

## Results

### ID1 and ID3 are upregulated in pulmonary fibrosis

**Figure 1:**
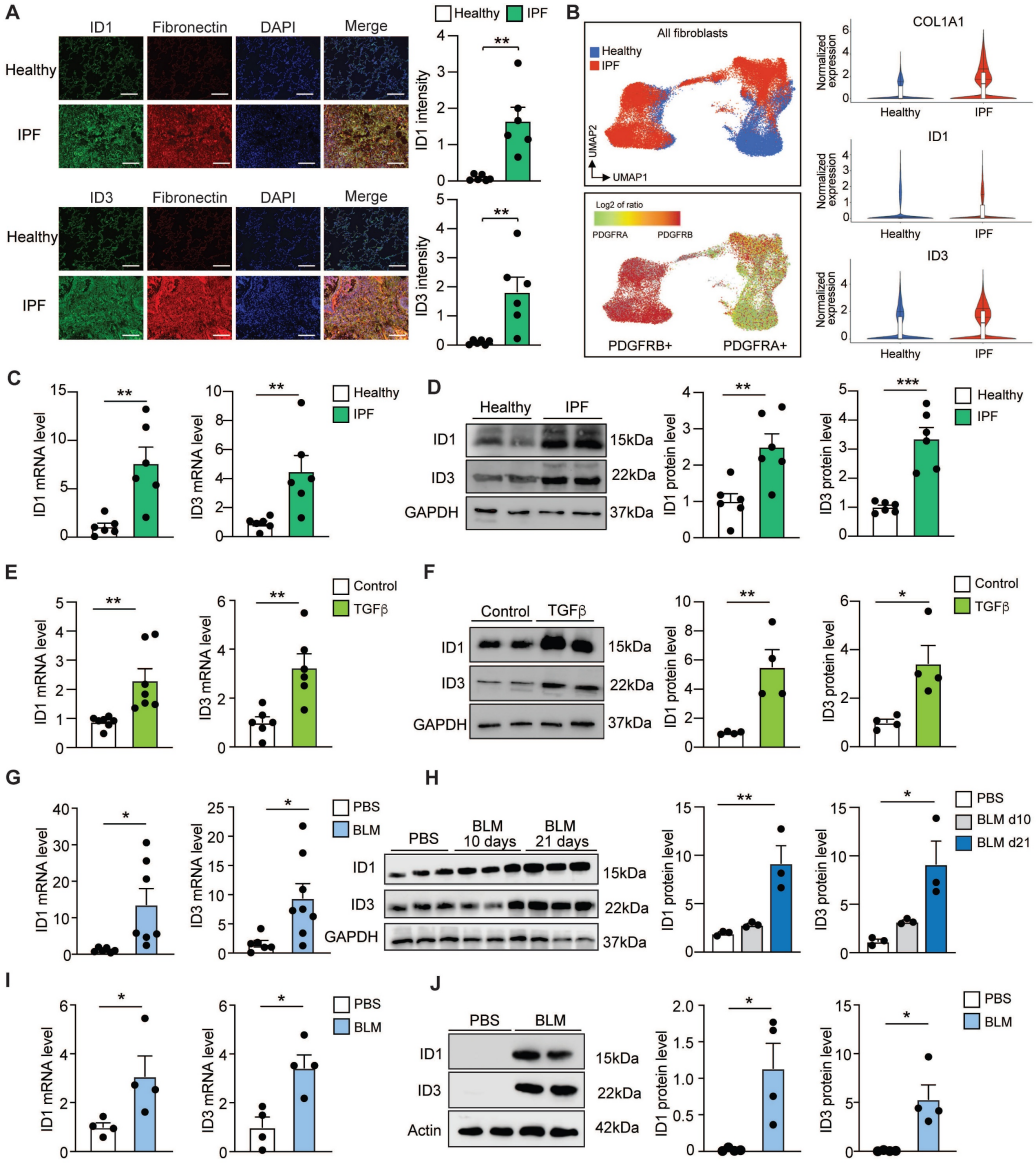
Increased ID1 and ID3 expression in pulmonary fibrosis. (A) (Left) Representative immunofluorescence images of lung sections from healthy donors and IPF patients showing ID1/ID3 (green) and Fibronectin (red) co-staining. Nuclei were counterstained with DAPI (blue). Scale bar: 100 µm. (Right) Quantification of the data. n = 6 patients per group. (B) (Left) UMAP plots showing the distribution of COL1A1⁺ fibroblasts in healthy and IPF samples, along with PDGFRA and PDGFRB expression. (Right) Violin plots showing the expression levels of Col1a1, ID1, and ID3 in fibroblasts from healthy and IPF lungs. (C-D) ID1 and ID3 mRNA (C) and protein (D) levels determined by real time qPCR and Western blot analyses in lung fibroblasts isolated from healthy donors and patients with IPF (n = 6/group). (E) ID1 and ID3 mRNA levels in healthy human lung fibroblasts treated with TGF-β1 (5 ng/ml) for 48 hours. n = 6-7 experiments performed in triplicate. (F) ID1 and ID3 protein levels in healthy human lung fibroblasts treated with TGF-β1 (5 ng/ml) for 48 hours. n = 4. (G) ID1 and ID3 mRNA levels in lungs of mice with pulmonary fibrosis (n = 6-8 mice/group). (H) ID1 and ID3 protein levels in lung homogenates 10 and 21 days after PBS or BLM injection (n = 3 mice/group). (I) ID1 and ID3 mRNA levels in lung fibroblasts isolated from the lungs of PBS or BLM-treated mice (n = 4 mice/group). (J) ID1 and ID3 protein levels in lung fibroblasts from control and BLM-treated mice (n = 4 mice/group). * P < 0.05, ** P < 0.01, by t-test for all panels, except for (H), which was analyzed using one-way-ANOVA.

To investigate the regulation of ID family members in pulmonary fibrosis, we first examined ID1 and ID3 expression in the lungs of patients with IPF and matched healthy donors. We found that ID1 and ID3 expression was significantly increased in the IPF lungs (Figure 1A). To identify the cell types in which ID1 and ID3 expression is increased in IPF, we next analyzed a publicly available single-cell RNA sequencing dataset from healthy and IPF lungs (GSE132771) 30. ID1 and ID3 levels were significantly elevated in fibroblasts from IPF patients (Figure 1A and Figure S1A). ID1 and ID3 expression was specifically increased in alveolar fibroblasts, adventitial fibroblasts, fibrotic fibroblasts (ID1), and peribronchial fibroblasts (ID3) from IPF patients (Figure S1B). Although ID1 and ID3 are also expressed in epithelial, endothelial, and immune cells, their expression was reduced in these cell types in IPF (Figure S1C), indicating that the overall increase in ID1 and ID3 expression in IPF lungs is predominantly attributable to lung fibroblasts.

We next quantified ID1 and ID3 mRNA levels in human lung fibroblasts (HLF) isolated from healthy donors and from patients with IPF. The PCR analyses revealed a significant increase in ID1 and ID3 mRNA levels in IPF-derived fibroblasts (Figure 1C). Western blot analyses of protein extracts from HLFs isolated from healthy donors and from patients with IPF revealed a marked upregulation of ID1 and ID3 in IPF-derived lung fibroblasts (Figure 1D). HLFs were then treated with TGF-β, the primary driver of pulmonary fibrosis 31, and ID1 and ID3 levels were determined by qPCR and Western blot. Our analyses revealed a prominent increase of ID1 and ID3 mRNA and protein levels in TGF-β-treated fibroblasts (Figure 1E-F). Next, we explored ID1/ID3 mRNA expression profiles in an *in vivo* animal model of lung fibrosis, bleomycin (BLM)-induced pulmonary fibrosis in mice. qPCR analyses revealed a significant increase in pulmonary ID1 and ID3 mRNA levels in diseased mice (Figure 1G). Consistent with the qPCR results, ID1 and ID3 protein levels were also increased in the lungs of mice three weeks after BLM administration (Figure 1H). Additionally, we examined the mRNA expression profiles of ID1 and ID3 in fibroblasts isolated from the lungs of mice with pulmonary fibrosis. qPCR and Western blot analyses revealed a significant increase in ID1 and ID3 mRNA and protein levels in fibroblasts isolated from diseased lungs (Figure 1I-J). Importantly, no significant changes in ID2 and ID4 levels were observed in lung fibroblasts from IPF patients, in healthy lung fibroblasts treated with TGF-β, or in the lungs of mice with pulmonary fibrosis (Figure S2A-C). These results indicate that ID1 and ID3 levels are increased in lung fibroblasts from mice and humans with pulmonary fibrosis, suggesting a contribution of these proteins to disease progression.

### Silencing of ID1 and ID3 attenuates human lung fibroblast proliferation, migration and differentiation into myofibroblast

**Figure 2:**
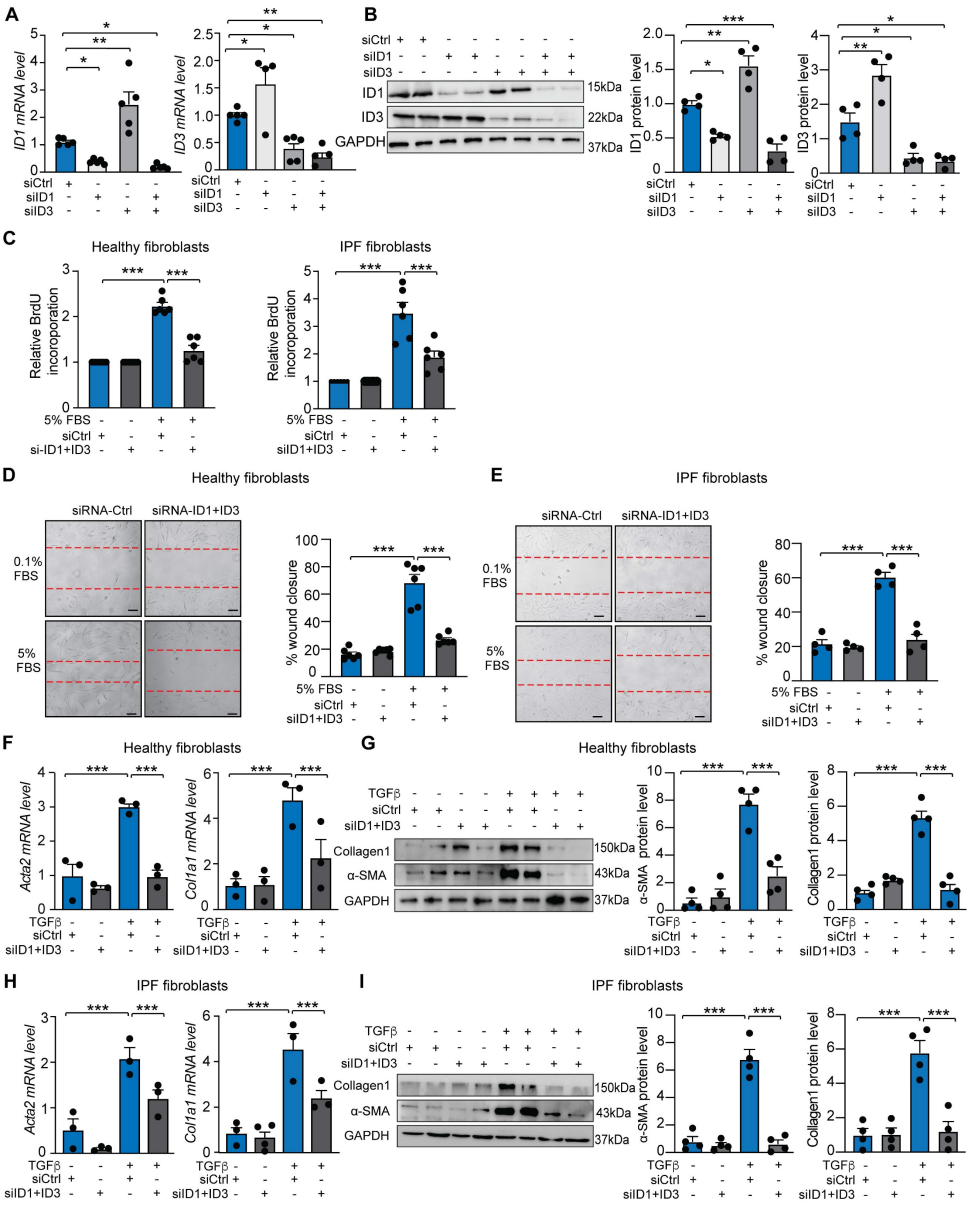
Knockdown of ID1/ID3 reduces human lung fibroblast proliferation, migration, and differentiation into myofibroblasts. (A) ID1 and ID3 mRNA levels in healthy human lung fibroblasts transfected with siRNA-Ctrl, siRNA-ID1 or siRNA-ID3 (5nM each) for 48 hours. n = 4-5 experiments performed in triplicate. (B) (Left) Representative immunoblots showing ID1, ID3 and GAPDH protein levels in healthy human lung fibroblasts transfected with siRNA-Ctrl, siRNA-ID1 or siRNA-ID3 (5nM each) for 48 hours. (Right) Quantification of the data. n = 4. (C) Proliferation of healthy and IPF-diseased human lung fibroblasts in the presence of the indicated treatments. n = 6 experiments performed in triplicate. (D-E) Migration of healthy (D) and IPF-derived (E) human lung fibroblasts in the presence of the indicated treatments. n = 4-6 experiments performed in duplicate. Scale bar: 100 µm. (F) qPCR assessment of Acta2 and Col1a1 mRNA levels in healthy HLF treated with siRNA-Ctrl or siRNA-ID1ID3 in the absence or presence of TGF-β1 (5 ng/ml). n = 3 experiments performed in triplicate. (G) (Left) Representative immunoblots showing Collagen1, α-SMA and GAPDH protein levels in healthy human lung fibroblasts transfected with siRNA-Ctrl or siRNA-ID1ID3 in the absence or presence of TGF-β1 (5 ng/ml). (Right) Quantification of the data. n = 4. (H) qPCR assessment of Acta2 and Col1a1 mRNA levels in IPF-derived HLF treated with siRNA-Ctrl or siRNA-ID1ID3 in the absence or presence of TGF-β1 (5 ng/ml). n = 3 experiments performed in triplicate. (G) (Left) Representative immunoblots showing Collagen1, α-SMA and GAPDH protein levels in IPF-derived HLFs transfected with siRNA-Ctrl or siRNA-ID1ID3 in the absence or presence of TGF-β1 (5 ng/ml). (Right) Quantification of the data. n = 4. * P < 0.05, ** P < 0.01, *** P < 0.001 by one-way ANOVA (A and B) or two-way ANOVA (C-I).

Given that lung fibroblasts contribute to injury responses through rapid migration, proliferation, and differentiation into myofibroblasts, we assessed whether ID1/ID3 inhibition affects these processes in HLFs. Knockdown of ID1 using a specific siRNA induced compensatory upregulation of ID3, and conversely, increased ID1 levels compensated for ID3 knockdown (Figure 2A-B), indicating that a simultaneous inhibition of ID1 and ID3 is necessary to accurately characterize their roles in pulmonary fibrosis. Combined knockdown of ID1/ID3 significantly reduced serum-induced proliferation in both healthy and IPF-derived HLFs (Figure 2C). We next examined whether ID1/ID3 inhibition affects HLF migration. Serum treatment increased migration in healthy and IPF-derived lung fibroblasts, whereas ID1/ID3 knockdown significantly attenuated this effect (Figure 2D-E). Because fibroblasts differentiate into myofibroblasts to enhance collagen production and promote fibrosis, we then investigated whether ID1/ID3 inhibition influences this process. Healthy HLFs were treated with TGF-β1 in the presence or absence of ID1 and ID3 specific siRNAs. As markers of fibroblasts differentiation, TGF-β1 increased alpha smooth muscle actin (Acta2) mRNA level, as well as Collagen 1a1 (Col1a1) mRNA levels (Figure 2F). Importantly, ID1/ID3 knockdown prevented these changes (Figure 2F), indicating that ID1/ID3 inhibition impairs lung fibroblast differentiation. Consistently, ID1/ID3 knockdown also reduced TGFβ-induced Acta2 and Collagen1 protein expression (Figure 2G). Similar effects were observed in IPF-derived HLFs (Figure 2H-I). Together, these results demonstrate that ID1/ID3 knockdown decreases lung fibroblast proliferation, migration and differentiation into myofibroblast.

We next investigated the effects of ID1/ID3 overexpression on HLF proliferation, migration and differentiation into myofibroblast. HLFs isolated from healthy human donors were transduced with adenoviruses encoding ID1 and ID3 (Ad-ID1+Ad-ID3) or with an adenovirus encoding luciferase (Ad-Ctrl) as a control. A significant increase in ID1 and ID3 mRNA levels, but not ID2 or ID4, was observed in Ad-ID1/ID3-treated HLFs (Figure S3A). Consistently, transduction with Ad-ID1/ID3 induced a marked increase in ID1 and ID3 protein levels (Figure S3B), confirming the efficiency of the adenoviral constructs. ID1/ID3 overexpression did not affect proliferation of healthy HLF but significantly increased fibroblast migration (Figure S3C-D). Importantly, ID1/ID3 overexpression induced Acta2, Col1a1 and Ctgf mRNA levels (Figure S3E) to levels comparable to those observed following TGF-β1 treatment (Figure 2). Together, these results suggest that ID1/ID3 overexpression is sufficient to enhance lung fibroblast migration, promote differentiation into myofibroblasts, and increase collagen production.

### Genetic deletion of ID1/ID3 protects mice from BLM-induced pulmonary fibrosis

**Figure 3:**
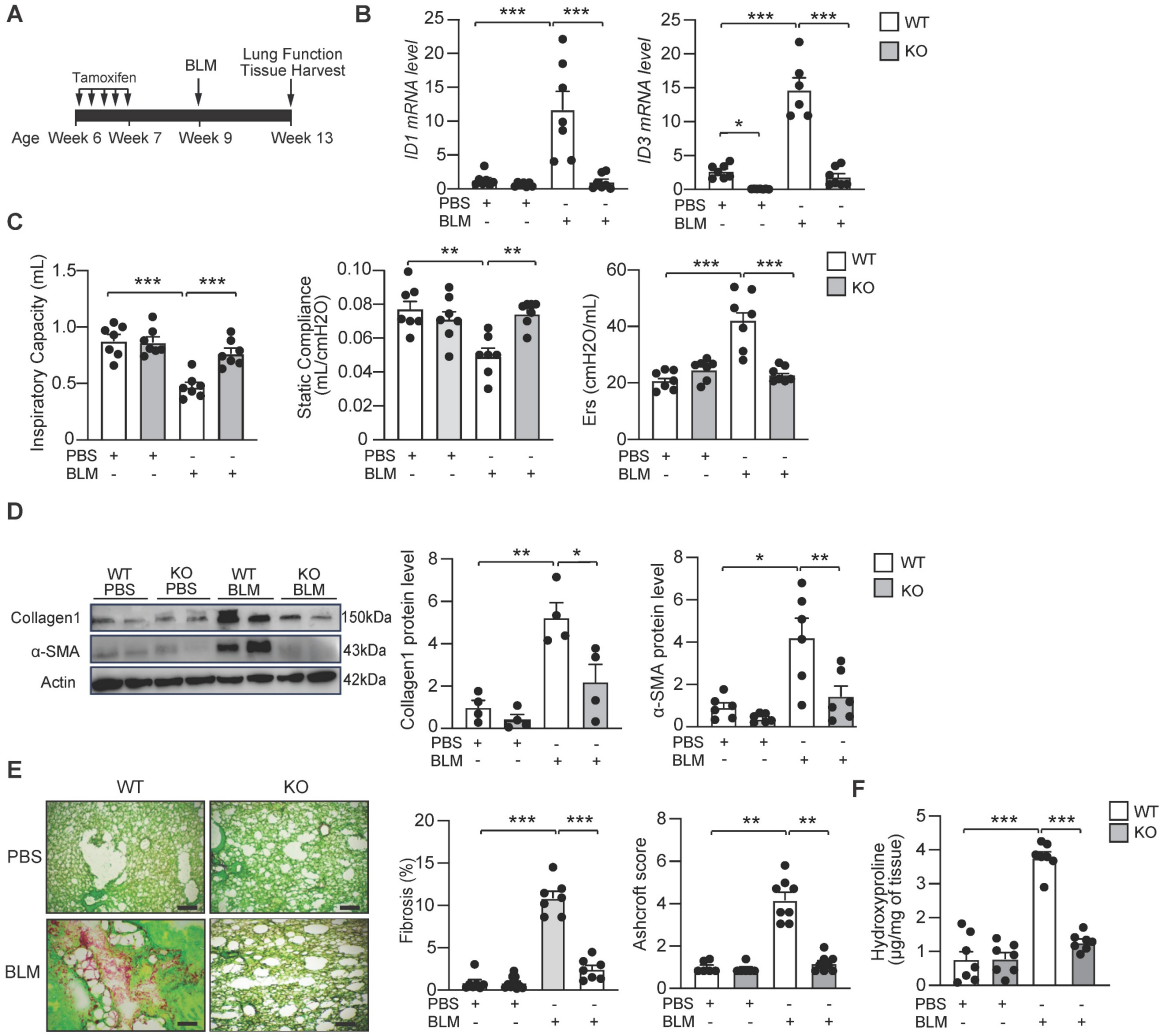
Genetic deletion of ID1 and ID3 reduces bleomycin-induced lung fibrosis. (A) Design of the study. (B) PCR analysis of ID1 and ID3 mRNA levels in lungs of the indicated groups. n=6-7 mice/group. (C) Lung function parameters, including inspiratory capacity, compliance and single frequency elastance. n=7 mice/group. (D) Lung protein expression of Collagen1 and α-SMA in lungs from the indicated groups. n=4-6 mice/group. (E) (Left) Representative images from Fast Green/Sirius-Red-stained lungs of the indicated groups. n=7 mice/group. Scale bars: 200 μm. (Middle) Quantitative analysis of fibrosis. (Right) Ashcroft scores representing the extent of fibrosis. n= 6-7 mice/group. (F) Hydroxyproline content in lungs from the indicated groups. n = 7 mice/group. * P < 0.05; ** P < 0.01. *** P < 0.001 by two-way ANOVA for all panels, except for the Ashcroft score, which was analyzed using the Kruskal-Wallis test followed by Dunn's multiple-comparison test.

To evaluate the *in vivo* effect of ID1/ID3 deletion on pathological remodeling in lung fibrosis, we generated ID1/ID3 double knockout mice. Because constitutive ID1/ID3 double knockout mice die at mid-gestation, we generated a fibroblast-specific conditional knockout model. Specifically, we globally ablated ID3 and conditionally deleted ID1 in fibroblasts using Col1a2-CreER mice. Mice received daily intraperitoneal (IP) injections of tamoxifen for five consecutive days (Figure 3A). Mice were then administered a single intratracheal dose of BLM (2U/kg) or saline and were sacrificed four weeks after BLM delivery (Figure 3A). As expected, BLM treatment in WT mice resulted in a marked increase in ID1 and ID3 expression, whereas ID1/ID3 KO mice exhibited significantly reduced pulmonary ID1 and ID3 mRNA levels (Figure 3B). Importantly, no significant changes in ID2 or ID4 expression were observed in the lungs of the ID1/ID3 KO mice (Figure S4A-B). Because disease progression in patients with IPF is commonly assessed by imaging and lung function analyses 32, and therapeutic efficacy is largely determined by improvements in lung function, we performed pulmonary function testing in these mice. BLM-exposed ID1/ID3 KO mice showed significant improvement in all parameters that were impaired in BLM-treated WT mice, including increased inspiratory capacity and static compliance, and decreased respiratory elastance (Figure 3C). Immunoblot analyses of lung homogenates revealed increased Collagen-I and α-SMA protein levels in BLM-treated WT mice, whereas ID1/ID3 KO mice displayed significantly reduced expression of these proteins (Figure 3D). Similarly, Col3a1 and Ctgf mRNA levels were elevated in response to BLM in WT mice but were significantly reduced in the lungs of ID1/ID3 KO mice (Figure S4C-D). Histological examination using Sirius Red/Fast Green staining demonstrated marked fibrosis in BLM-treated WT mice, whereas ID1/ID3 KO mice exhibited substantially reduced fibrotic remodeling (Figure 3E and S5A). Consistently, ID1/ID3 KO mice had significantly lower Ashcroft scores compared with WT mice (Figure 3E). Masson's trichrome staining further confirmed reduced fibrosis and lower Ashcroft scores in ID1/ID3 KO mice (Figure S6A). Finally, hydroxyproline content was significantly decreased in ID1/ID3 KO mice compared with BLM-treated WT mice (Figure 3F). Taken together, these findings indicate that deletion of ID1/ID3 prior to BLM exposure protects mice from the development of lung fibrosis.

### Pharmacological inhibition of ID1/ID3 protects mice from pulmonary fibrosis

**Figure 4:**
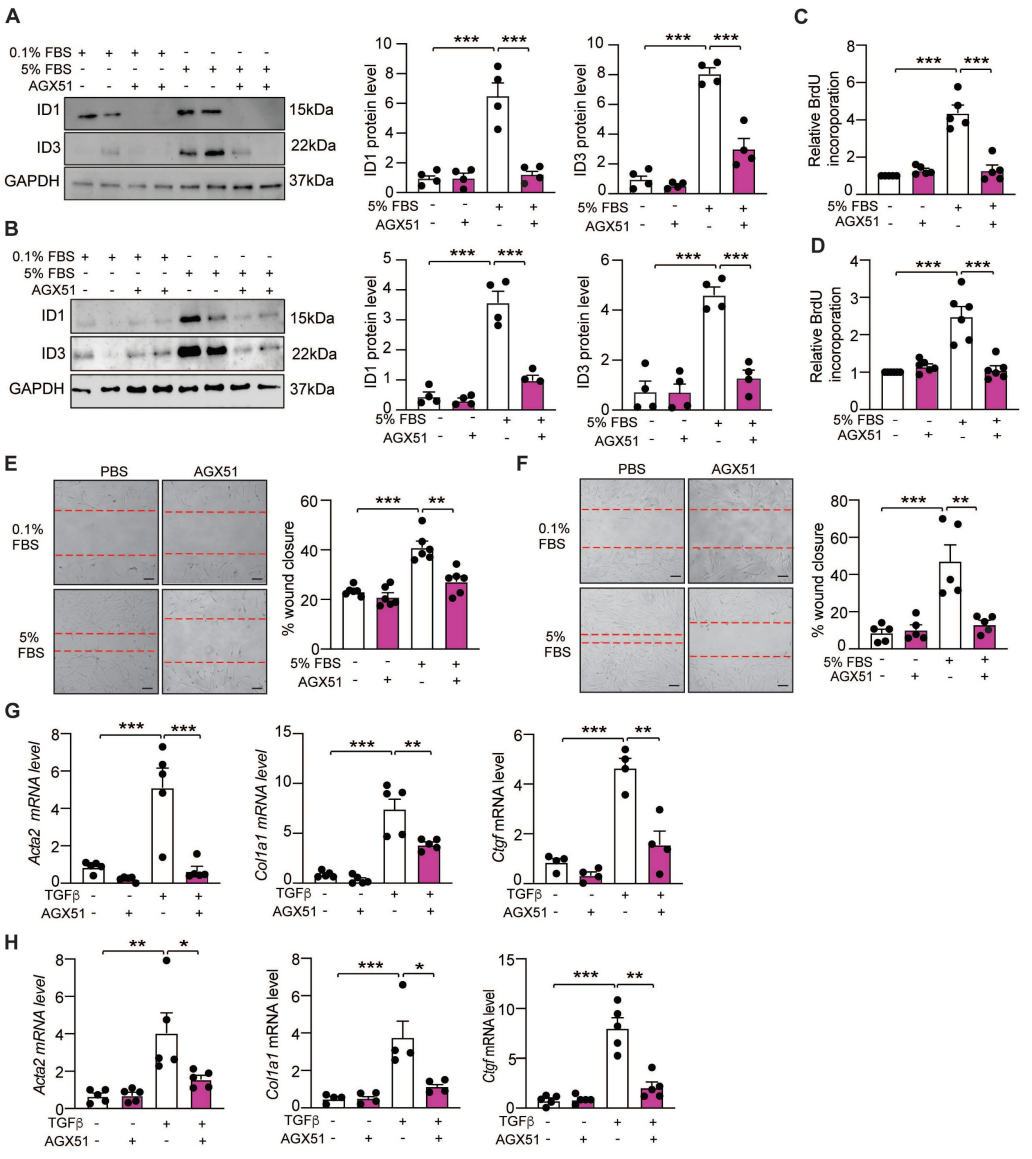
Pharmacological inhibition of ID1/ID3 reduces lung fibroblast proliferation, migration, and differentiation into myofibroblast. (A-B) (Left) Representative immunoblots showing ID1, ID3 and GAPDH protein levels in healthy (A) and IPF-derived (B) human lung fibroblasts treated with 0.1%FBS or 5%FBS in the presence or absence of an ID1/ID3 pharmacological inhibitor (AGX51, 20µM). (Right) Quantification of the data. n = 4. (C-D) Proliferation of healthy (C) and IPF-derived (D) human lung fibroblasts in the presence or absence of AGX51 (20µM). n = 5-6 experiments performed in triplicate. (E-F) Migration of healthy (E) and IPF-derived (F) human lung fibroblasts in the presence or absence of AGX51 (20µM). n = 5-6 experiments performed in triplicate. Scale bar: 100 µm. (G-H) qPCR assessment of Acta2, Col1a1 and Ctgf mRNA levels 48h after healthy (G) and IPF-derived (H) human lung fibroblasts treatment with TGF-β1 (5 ng/ml) and AGX51 (20 µM). n = 4-5 experiments performed in triplicate. * P < 0.05; ** P < 0.01. *** P < 0.001 by two-way ANOVA.

We next assessed whether a pharmacological inhibitor of ID1 and ID3 (AGX51) affects HLFs proliferation, migration and differentiation into myofibroblasts. This small molecule promotes ubiquitin-mediated degradation of ID1 and ID3 in both healthy and IPF-derived HLFs, as demonstrated by reduced ID1 and ID3 expression in AGX51-treated cells (Figure 4A-B). Consistent with the results obtained using ID1- and ID3-specific siRNAs, AGX51 inhibited serum-induced proliferation of both healthy and IPF-derived HLFs (Figure 4C-D). Similarly, AGX51 treatment significantly reduced migration of healthy and IPF-derived HLFs (Figure 4E-F). In addition, AGX51 attenuated the differentiation of both healthy and IPF-derived HLFs, as assessed by qPCR and immunoblot analyses of fibrotic markers (Figure 4G-H and S7). Importantly, AGX51 treatment did not exert additional effects in cells in which ID1 and ID3 were already silenced (Figure S8), indicating that the biological effects of AGX51 are mediated specifically through ID1/ID3 inhibition.

Next, to assess the potential involvement of ID1/ID3 in epithelial-mesenchymal transition (EMT), primary human alveolar epithelial cells (hAEpiCs) were treated with TGF-β in the presence or absence of AGX51. Pharmacological inhibition of ID1/ID3 attenuated TGF-β-induced upregulation of mesenchymal and fibrotic markers, including Vimentin, Fibronectin, Col3A1, and Snail1 (Figure S9). Conversely, overexpression of ID1/ID3 enhanced TGF-β-induced expression of these markers (Figure S9). These results suggest that ID1/ID3 modulate epithelial plasticity and EMT-related responses under profibrotic conditions.

**Figure 5:**
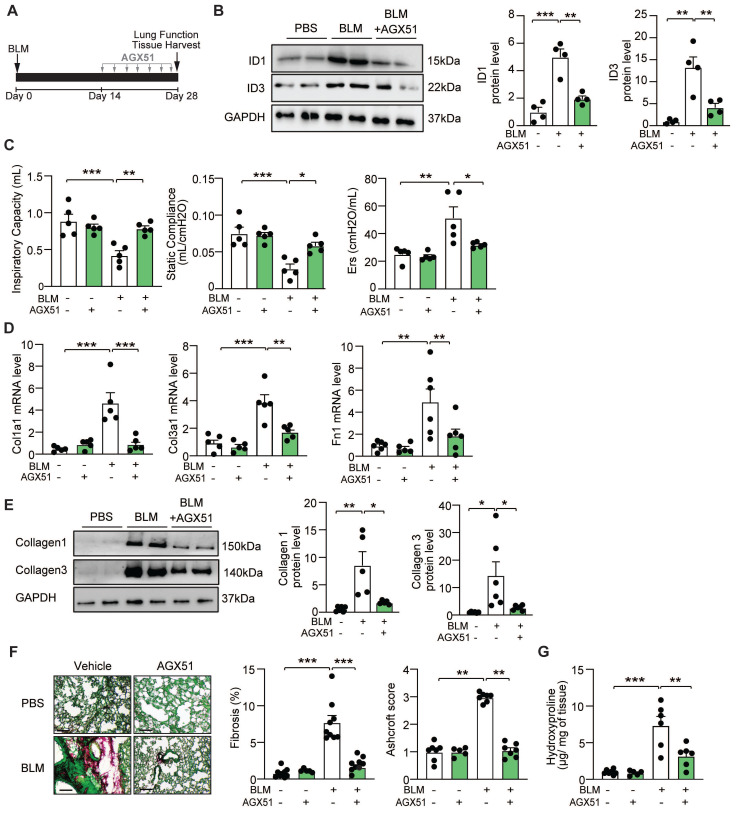
Pharmacological inhibition of ID1/ID3 confers protection against pulmonary fibrosis in mice. (A) Design of the study. (B) (Left) Representative immunoblots showing ID1, ID3 and GAPDH protein levels in lung homogenates from PBS- or BLM-challenged mice treated or not with AGX51. (Right) Quantification of the data. n = 4 mice/group. (C) Lung function parameters, including inspiratory capacity, compliance, and single frequency elastance. n=5 mice/group. (D) PCR analysis of Col1a1, Col3a1 and Fn1 mRNA levels in lungs of the indicated groups. n=5-6 mice/group. (E) Lung protein expression of Collagen-I and Collagen-III in lungs from the indicated groups. n=5-6 mice/group. (F) (Left) Representative images from Fast Green/Sirius-Red-stained lungs of the indicated groups. n=5-9 mice/group. Scale bars: 200μm. (Middle) Quantitative analysis of fibrosis. (Right) Ashcroft scores representing the extent of fibrosis. n = 5-7 mice/group. (G) Hydroxyproline content in lungs from the indicated groups. n = 5-7 mice/group. * P < 0.05; ** P < 0.01. *** P < 0.001 by one-way ANOVA (B and E), two-way ANOVA (C, D, F, and G), and by Kruskal-Wallis test followed by Dunn's multiple-comparison test for the Ashcroft score.

To assess the *in vivo* effects of AGX51 on pulmonary fibrosis, WT mice were intratracheally administered BLM (2U/kg) or saline, followed by two weeks of AGX51 treatment (50 mg/kg, twice daily, three days per week) starting on day 14 from BLM administration (Figure 5A). BLM induced a marked increase in ID1 and ID3 expression levels, which was significantly reduced by AGX51 treatment (Figure 5B). AGX51 treatment alone had no effect in non-fibrotic mice (Figure 5C-G). AGX51 treatment improved lung function in BLM-treated mice, as evidenced by increased respiratory capacity and static compliance, and reduced respiratory elastance (Figure 5C). The mRNA expression levels of Col1a1, Col3a1, and fibronectin (*Fn1*) were significantly reduced in the lungs of BLM-challenged mice treated with AGX51 (Figure 5D). Immunoblot analysis of lung homogenates further demonstrated a marked decrease in collagen I and collagen III protein levels following AGX51 treatment (Figure 5E). Histological examination using Sirius Red/Fast Green staining revealed extensive fibrosis in BLM-treated mice, whereas AGX51 administration markedly attenuated fibrotic remodeling (Figure 5F and S5B). Consistently, AGX51-treated mice exhibited significantly lower Ashcroft scores compared with PBS-treated controls (Figure 5F and S6B). In addition, hydroxyproline content was significantly reduced in AGX51-treated mice (Figure 5G). Taken together, these findings indicate that pharmacological inhibition of ID1 and ID3 attenuates the development of BLM-induced lung fibrosis in mice.

We next compared the effects of ID1/ID3 inhibition with those of currently approved antifibrotic therapies. Mice were administered bleomycin and, two weeks later, treated with AGX51, pirfenidone, or nintedanib for an additional two weeks (Figure S10A). AGX51 reduced collagen deposition and histological fibrosis scores to a degree comparable to pirfenidone and nintedanib (Figure S10B-C, S5D, and S6D). Importantly, AGX51 produced statistically significant improvements in selected lung function parameters compared not only with vehicle-treated mice, but also with pirfenidone- and nintedanib-treated groups (Figure S10D). These findings suggest that, in this experimental model, AGX51 may confer greater functional benefit than currently approved antifibrotic therapies.

Because fibroblast senescence has been implicated in IPF pathogenesis and ID1 has been described as a regulator of this process, we next determined whether modulation of ID1/ID3 affects cellular senescence in the context of pulmonary fibrosis. Senescence-associated β-galactosidase (SA-β-gal) staining was significantly increased in the lungs of BLM-treated WT mice, consistent with enhanced cellular senescence during pulmonary fibrosis (Figure S11A). Importantly, genetic deletion of ID1/ID3 significantly attenuated the BLM-induced increase in SA-β-gal activity (Figure S11A). Similarly, pharmacological inhibition of ID1/ID3 significantly reduced BLM-induced SA-β-gal staining (Figure S11B). These findings indicate that inhibition of ID1/ID3 suppresses fibrosis-associated cellular senescence *in vivo*.

### Targeted inhibition of ID1/ID3 in the lung attenuates BLM-induced pulmonary fibrosis

**Figure 6:**
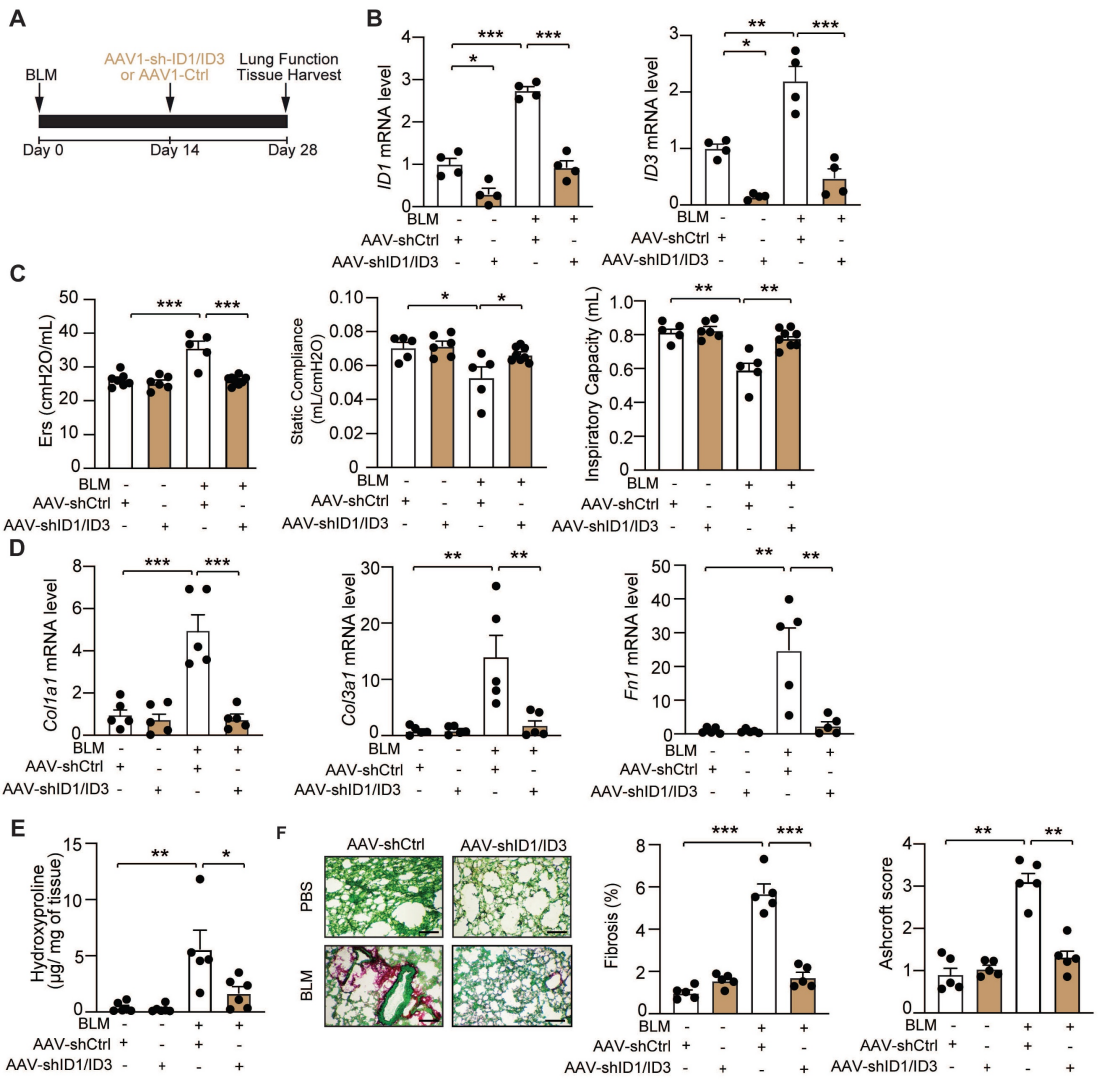
Lung-specific inhibition of ID1 and ID3 attenuates bleomycin-induced lung fibrosis. (A) Schematic representation of the study design. (B) qPCR analysis of ID1 and ID3 mRNA levels in lungs of the indicated groups. n = 4 mice/group. (C) Lung function parameters, including inspiratory capacity, static compliance, and single-frequency elastance. n = 5-8 mice/group. (D) Relative mRNA expression of Col1a1, Col3a1, and Fn1 in lungs from the indicated groups. n=5 mice/group. (E) Hydroxyproline content in lungs from the indicated groups. n=5-6 mice/group. (F) (Left) Representative Fast Green/Sirius-Red-stained lung sections of the indicated groups. n=5-mice/group. Scale bar: 200μm. (Middle) Quantitative analysis of fibrosis. (Right) Ashcroft scores reflecting the extent of fibrosis. n = 5 mice/group. * P < 0.05; ** P < 0.01. *** P < 0.001 by two-way ANOVA for all panels, except for the Ashcroft score, which was analyzed using the Kruskal-Wallis test followed by Dunn's multiple-comparison test.

To selectively modulate ID1 and ID3 expression in the lung, we generated adeno-associated viruses 1 (AAV1) expressing short hairpins targeting ID1 and ID3 (shID1 and shID3) and delivered them via intratracheal injection to mice two weeks after BLM administration (Figure 6A). BLM induced a significant increase in ID1 and ID3 mRNA levels in AAV1-Ctrl-treated mice, whereas AAV1-shID1/ID3 treatment markedly reduced pulmonary ID1 and ID3 mRNA expression (Figure 6B). Intratracheal delivery of AAV-shID1/ID3 did not significantly alter ID1 and ID3 mRNA levels in the heart, liver, or kidney (Figure S12), indicating lung-restricted effects. Under basal conditions, intratracheal administration of AAV1-shID1/ID3 did not produce detectable phenotypic changes (Figure 6C-F). In BLM-treated mice, however, AAV1-shID1/ID3 delivery improved lung function, as evidenced by increased inspiratory capacity and static compliance, and decreased respiratory elastance (Figure 6C). AAV1-shID1/ID3-treated mice exhibited significantly reduced Col1a1, Col3a1, and Fn1 mRNA levels (Figure 6D). In addition, lung hydroxyproline content was significantly decreased (Figure 6E). Histological analysis using Sirius Red/Fast Green staining revealed marked fibrosis in AAV1-Ctrl-treated mice, whereas AAV1-shID1/ID3 administration significantly attenuated fibrotic remodeling (Figure 6F and S5C). Consistently, AAV1-shID1/ID3-treated mice displayed lower Ashcroft score compared with AAV1-Ctrl-treated mice (Figure 6F and S6C). Together, these findings show that lung-specific inhibition of ID1 and ID3 attenuates BLM-induced pulmonary fibrosis.

### Transcriptional regulation of ID1 and ID3 by Egr-1 and hypoxia in lung fibroblasts

By analyzing transcription factor binding sites within the promoter regions of ID1 and ID3 using the PROMO v3.0.2 database 33, we identified Egr-1 as a predicted regulator of both genes. Egr-1 expression has been reported to be increased in IPF 34 and implicated in pulmonary fibrosis 35. Overexpression of Egr1 significantly increased ID1 and ID3 mRNA (Figure S13A) and protein (Figure S13B) levels. Given that hypoxia has been implicated in the pathogenesis of fibrotic diseases and linked to the development of IPF 36, 37, we next investigated whether hypoxia and hypoxia-inducible factor 1α (HIF-1α) regulate ID1 and ID3 expression. HLFs were transfected with HIF-1α-specific siRNA or control siRNA and subsequently exposed to normoxic (21% O₂) or hypoxic (1% O₂) conditions. Hypoxia induced a significant upregulation of ID1 and ID3 expression, an effect that was abolished by HIF-1α knockdown (Figure S13C).

### ID and ID3 regulate cell cycle-related genes and signal through the MEK/ERK pathway

**Figure 7:**
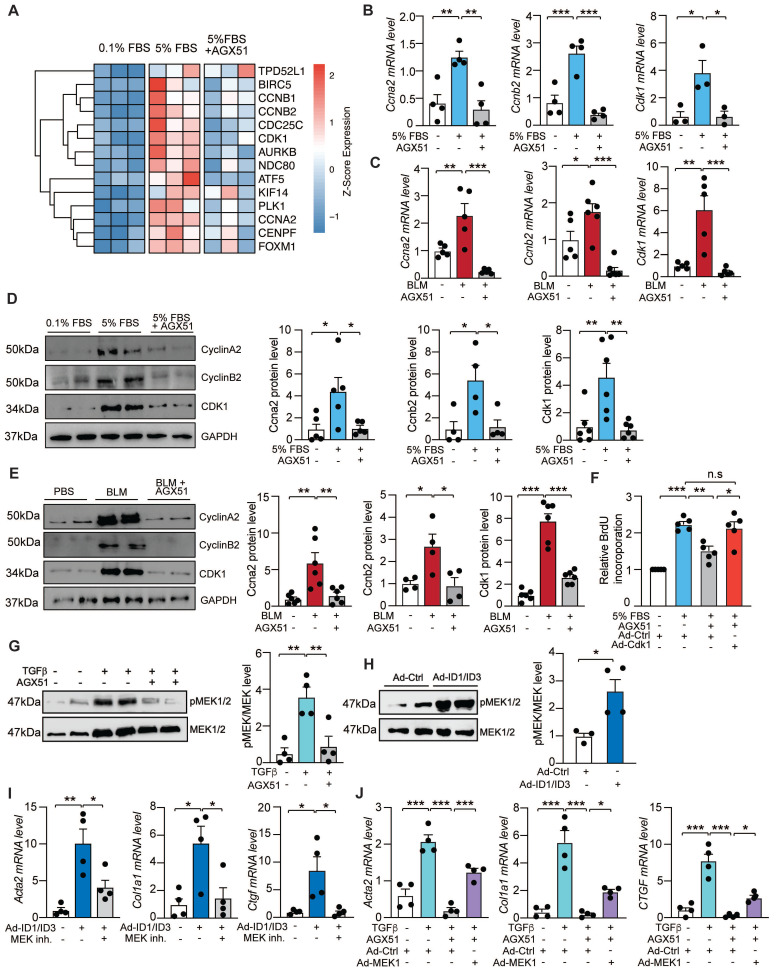
ID1/ID3 act through modulation of cell cycle-associated genes and activation of the MEK signaling pathway. (A) Heat map of differentially expressed cell cycle-related genes from the Gene Ontology Biological Process pathway 'cell cycle G2/M phase transition' (GO:0044839) in PBS-treated versus AGX51-treated HLFs cultured in the presence of 0.1% FBS or 5% FBS. (B) PCR analysis of Ccna2, Ccnb2 and Cdk1 mRNA levels in HLF treated with PBS or AGX51 in the presence of Serum (5% FBS). n = 3-4 per group. (C) Pulmonary Ccna2, Ccnb2 and Cdk1 mRNA levels in lungs from the indicated groups. n = 5-6 per group. (D) (Left) Representative immunoblots showing CCNA2, CCNB2 and CDK1 protein levels in HLFs treated with 5%FBS in the presence or absence of AGX51. (Right) Quantification of protein expression. n = 4-6 per group. (E) (Left) Representative immunoblots showing CCNA2, CCNB2 and CDK1 protein levels in lung homogenates from the indicated groups. (Right) Quantification of protein expression. n = 4-6 per group. (F) Proliferation of human lung fibroblasts under the indicated treatment conditions. n = 5 experiments performed in triplicate. (G) Representative immunoblots showing phosphorylated MEK1 (p-MEK1) and total MEK1 protein levels in HLF treated with 5%FBS in the presence or absence of AGX51. (Right) Quantification of the data. n = 4 per group. (H) Representative immunoblots showing phosphorylated MEK1 and total MEK1 protein levels in HLF treated with Adenovirus control (Ad-Ctrl) or Adenoviruses ID1+ID3 (Ad-ID1/ID3). (Right) Quantification of the data. n = 3-4 per group. (I) qPCR analysis of Acta2, Col1a1, and Ctgf mRNA levels 48h after HLF treatment with Ad-ID1/ID3 or Ad-Ctrl in the presence or absence of the MEK1 inhibitor Selumetinib (10 µM). n = 4 experiments performed in duplicate. (J) qPCR analysis of Acta2, Col1a1, and Ctgf mRNA levels 48h after HLF treatment with Ad-MEK1 or Ad-Ctrl in the presence or absence of AGX51 (20 µM). n = 4 experiments performed in duplicate. * P < 0.05; ** P < 0.01. *** P < 0.001 by one-way ANOVA for all panel, expect for (H) which was analyzed using a t-test.

To elucidate the functional roles of ID1 and ID3 in pulmonary fibrosis, it is critical to identify their mechanism(s) of action. To this end, we performed RNA sequencing analysis on RNA extracted from human lung fibroblasts treated with the ID1 and ID3 inhibitor (AGX51) or PBS (control) in the presence of serum (5% FBS), using three biological replicates per group. Principal component analysis (PCA) of normalized read counts revealed clear separation between treatment groups (Figure S14A). Samples clustered distinctly according to treatment condition, with AGX51-treated cells clearly segregated from untreated cells (Figure S14A). Differential expression analysis identified multiple mRNAs and signaling pathways regulated by AGX51 (Figure S14B-C and Table S4), and transcription factor activity analysis predicted altered activity of several transcription factors in response to ID1/ID3 inhibition (Figure S15). Pathway enrichment analysis revealed that the majority of enriched pathways derived from upregulated genes in serum-treated HLFs were associated with cell cycle processes (Figure S14D-E). Notably, there was a consistent downregulation of the genes within these pathways with AGX51 treatment (Figure 7A). Among the differentially expressed genes were cyclin A2 (Ccna2), cyclin B2 (Ccnb2), and cyclin-dependent kinase 1 (Cdk1) (Figure 7A). To validate the RNA sequencing results, mRNA was isolated from AGX51-treated HLFs and analyzed by qPCR. AGX51 significantly reduced Ccna2, Ccnb2, and Cdk1 mRNA levels (Figure 7B). *In vivo*, BLM treatment induced marked upregulation of pulmonary Ccna2, Ccnb2, and Cdk1 mRNA expression, which was significantly reversed by pharmacological inhibition of ID1/ID3 (Figure 7C). Immunoblot analysis in serum-stimulated HLFs and lungs of BLM-treated mice confirmed increased protein levels of cyclin A2, cyclin B2, and CDK1, whereas ID1/ID3 inhibition reduced their expression (Figure 7D-E). These findings were further validated following genetic inhibition of ID1/ID3. Transfection of HLFs with ID1/ID3-specific siRNAs markedly reduced Ccna2, Ccnb2, and Cdk1 mRNA and protein levels (Figure S16A-B), and AAV-shID1/ID3 administration attenuated BLM-induced upregulation of these cell cycle proteins *in vivo* (Figure S16C-D).

We next performed a rescue experiment to validate the functional relevance of the cell cycle gene pathway in mediating the effects of ID1/ID3. HLFs were treated with AGX51 in the presence of an adenovirus encoding Cdk1 (Ad-Cdk1) or a control adenovirus. Transduction with Ad-Cdk1 significantly increased Cdk1 mRNA and protein levels (Figure S17A-B), confirming the efficiency of the adenoviral construct. Although ID1/ID3 inhibition reduced HLF proliferation, Cdk1 overexpression largely abrogated the anti-proliferative effect of AGX51 (Figure 7F).

Because ID1 and ID3 have been reported to regulate the MEK/ERK pathway in cancer 21 and MEK signaling is known to promote lung fibroblast differentiation into myofibroblast 38, we next assessed the effect of ID1/ID3 inhibition on MEK1 phosphorylation in HLFs. Western blot analysis revealed that ID1/ID3 inhibition decreased MEK1 phosphorylation in TGFβ1-treated HLFs (Figure 7G), whereas ID1/ID3 overexpression increased p-MEK1 levels (Figure 7H). Consistently, ID1/ID3 inhibition reduced ERK1/2 phosphorylation in TGFβ1-treated HLFs (Figure S18A), while ID1/ID3 overexpression increased phosphorylated ERK1/2 levels (Figure S18B). Elevated ID1/ID3 expression promoted fibroblasts differentiation into myofibroblasts, as indicated by increased Acta2, Col1a1 and Ctgf mRNA levels; however, MEK1 inhibition abrogated these effects (Figure 7I). Conversely, adenoviral overexpression of MEK1 in HLFs (Figure S17C) significantly attenuated the inhibitory effect of ID1/ID3 knockdown on fibroblast differentiation (Figure 7J).

Together, these findings indicate that ID1/ID3 regulate lung fibroblast proliferation through the Cyclin A2/Cyclin B2/CDK1 axis and promote fibroblast differentiation into myofibroblasts via activation of the MEK/ERK pathway.

## Discussion

Ongoing research aims to better understand the pathophysiology of IPF and to define new therapeutic strategies for this devastating disease. Because currently approved therapies do not fully halt disease progression, there remains a significant unmet medical need. In this study, we investigated the role of ID proteins in pulmonary fibrosis and evaluated their potential as therapeutic targets. ID proteins are highly conserved transcriptional regulators that play pivotal roles during developmental 39 and are typically low in adult tissues, although they are re-expressed in several cancers 40-44.

Although ID1 has been reported to be increased in the lungs of BLM-treated mice 45, 46, its regulation in human IPF and the expression of ID3 in pulmonary fibrosis had not been comprehensively examined. Here, we demonstrate that ID1 and ID3 mRNA levels are significantly elevated in lungs and lung fibroblasts from patients with IPF and from mice with pulmonary fibrosis. ID1 and ID3 were also upregulated in human lung fibroblasts following TGF-β1 treatment, a key profibrotic cytokine. These findings establish the clinical and experimental relevance of ID1/ID3 in IPF.

Mechanistically, we show that ID1/ID3 regulate key pathways involved in fibroblast proliferation, migration and differentiation into myofibroblast. Simultaneous inhibition of ID1/ID3 reduced cyclin A2, cyclin B2 and CDK1 expression, and CDK1 overexpression rescued the anti-proliferative effect of ID1/ID3 inhibition. In addition, ID1/ID3 overexpression enhanced MEK1 and ERK1/2 phosphorylation, whereas MEK1 inhibition abrogated ID1/ID3-induced fibroblast differentiation. Thus, ID1/ID3 promote fibroblast activation through regulation of cell cycle machinery and MEK/ERK signaling.

The role of MEK/ERK signaling in fibroblast biology is complex and context-dependent. While several studies demonstrate that MEK/ERK activation promotes lung fibroblast differentiation and extracellular matrix production in fibrotic settings 43, 47-49, other work shows that FGF2 can induce dedifferentiation of established myofibroblasts through MEK/ERK signaling 50. These differences likely reflect variation in upstream ligands, signal duration and magnitude, and fibroblast activation state. Our findings support a context-dependent model in which MEK/ERK contributes to profibrotic remodeling under TGF-β-driven conditions.

Previous studies have reported findings that appear to contrast with ours. For example, ID1 deletion promoted lung collagen accumulation 45, 46, ID3 overexpression has been associated with antifibrotic effects in fibroblasts 51 and ID1 or ID3 overexpression separately in skin fibroblasts reduced α-SMA and COL1A1 expression 52. These observations suggest that ID proteins may exert antifibrotic functions in certain contexts. However, several factors may account for these discrepancies. First, fibroblasts exhibit marked tissue-specific heterogeneity, and the functional role of ID1/ID3 may differ between skin and lung fibroblasts. Second, many prior studies relied on overexpression systems, which may induce supraphysiologic protein levels and non-physiological transcriptional programs, whereas our study interrogates the role of endogenous ID1/ID3 using genetic and pharmacologic inhibition. Third, ID1 and ID3 have overlapping and compensatory functions 53-56 and Figure 2A-B; therefore, single-gene manipulation may yield outcomes distinct from simultaneous inhibition. By targeting ID1 and ID3 concurrently *in vivo*, our study reveals a cooperative profibrotic role in the lung that may not be apparent in single-gene or gain-of-function systems. Together, these considerations support a context-dependent model in which the function of ID proteins is influenced by tissue type, cellular microenvironment, disease stage, and the mode of experimental manipulation.

Because complete germline deletion of both ID1 and ID3 results in embryonic lethality at mid-gestation 57, we generated mice carrying a ubiquitous deletion of ID3 combined with a fibroblast-specific deletion of ID1. This approach enabled effective ablation of ID signaling in fibroblasts while preserving viability and allowed us to demonstrate that simultaneous inhibition of ID1 and ID3 protects against the development of pulmonary fibrosis. Although we cannot fully exclude subtle systemic effects resulting from global ID3 deficiency, our *in vitro* fibroblast-specific loss- and gain-of-function studies strongly support a predominantly fibroblast-autonomous mechanism. In addition, we found that both genetic deletion and pharmacological inhibition of ID1/ID3 significantly attenuated the bleomycin-induced increase in cellular senescence. Whether this reduction in senescence reflects a direct effect of ID1/ID3 inhibition on senescence pathways or occurs secondary to the overall attenuation of pulmonary fibrosis remains to be determined.

Therapeutically, we demonstrate that both preventive and post-injury interventions targeting ID1/ID3 are effective. The genetic deletion of ID1/ID3 represents a preventive model in which gene inactivation precedes bleomycin exposure, thereby demonstrating that ID1/ID3 are required for the initiation and progression of fibrogenesis. In contrast, the pharmacologic (AGX51) and AAV-shRNA approaches were administered after fibrosis had been established, modeling a therapeutic setting. These post-injury interventions therefore address whether inhibition of ID1/ID3 can attenuate ongoing fibrotic remodeling and partially reverse established disease. Together, these complementary approaches support both a necessary role for ID1/ID3 in fibrosis development and their potential as therapeutic targets in established pulmonary fibrosis.

AGX51, a small-molecule pan-ID antagonist 58-60, phenocopied genetic ID1/ID3 loss, reversed profibrotic phenotypes *in vitro*, improved lung function, and reduced fibrosis *in vivo*. The selected dosing regimen was based on prior reports of good tolerability 61 and we observed no overt toxicity in treated animals. These findings support the feasibility of pharmacologic ID1/ID3 targeting. To further enhance translational relevance and reduce potential off-target effects, we developed intratracheal AAV1-shID1/ID3 delivery, which significantly improved lung function and reduced fibrosis. Future studies using fibroblast-specific promoters may further refine lung-targeted ID1/ID3 inhibition.

In summary, we propose a model in which ID1 and ID3 are upregulated in pulmonary fibrosis by key profibrotic stimuli, including TGF-β, hypoxia, and Egr-1, thereby integrating inflammatory and stress-related signaling pathways. Increased ID1/ID3 expression promotes fibroblast proliferation and differentiation through regulation of cell cycle genes and MEK/ERK signaling. Simultaneous inhibition of ID1/ID3 attenuates fibroblast activation and reduces pulmonary fibrosis *in vivo*, supporting ID1/ID3 as promising therapeutic targets in IPF.

## Supplementary Material

Supplementary figures and tables 1-3; Table S1 provides the characteristics of the human patients; Table S2 lists the primer sequences used for real-time qPCR; Table S3 provides the antibodies used for western blot analysis.

Supplementary table 4: Table S4 contains the RNA sequencing raw counts.

## Data Availability

All data reported in this paper will be shared by the corresponding author (Yassine Sassi) upon responsible request.

## Abbreviations

IPF: idiopathic pulmonary fibrosis
ECM: extracellular matrix
TGF-β: transforming growth factor-beta
ID: inhibitors of DNA binding
HLH: helix-loop-helix
HLF: human lung fibroblasts
MEK/ERK: mitogen-activated protein kinase/extracellular-signal-regulated kinase
AAV1: adeno-associated virus 1
CDK1: cyclin dependent kinase 1
FBS: fetal bovine serum
hFGF: human fibroblast growth factor
Egr-1: early growth response 1
Cdc2: cell division control protein 2
WT: Wild-type
KO: knockout
HIF-1α: Hypoxia-Inducible Factor-1 alpha
cDNA: complementary DNA
BLM: bleomycin
Acta2: alpha smooth muscle actin
Col1a1: Collagen 1a1
Ctgf: connective tissue growth factor
EMT: epithelial-mesenchymal transition
Fn1: Fibronectin
Ccna2: cyclin A2
Ccnb2: cyclin B2

## References

[1] Glass DS, Grossfeld D, Renna HA, Agarwala P, Spiegler P, DeLeon J (2022). Idiopathic pulmonary fibrosis: current and future treatment. Clin Respir J.

[2] Korfei M, Mahavadi P, Guenther A (2022). Targeting histone deacetylases in idiopathic pulmonary fibrosis: a future therapeutic option. Cells.

[3] Martinez FJ, Collard HR, Pardo A, Raghu G, Richeldi L, Selman M (2017). Idiopathic pulmonary fibrosis. Nat Rev Dis Primers.

[4] Luppi F, Kalluri M, Faverio P, Kreuter M, Ferrara G (2021). Idiopathic pulmonary fibrosis beyond the lung: understanding disease mechanisms to improve diagnosis and management. Respir Res.

[5] Joshi PR (2024). Pulmonary diseases in older patients: understanding and addressing the challenges. Geriatrics.

[6] Zhang D, Newton CA, Wang B, Povysil G, Noth I, Martinez FJ (2022). Utility of whole genome sequencing in assessing risk and clinically relevant outcomes for pulmonary fibrosis. Eur Respir J.

[7] Wagner DE, Alsafadi HN, Mitash N, Justet A, Hu Q, Pineda R (2025). Inhibition of epithelial cell YAP-TEAD/LOX signaling attenuates pulmonary fibrosis in preclinical models. Nat Commun.

[8] Jiang M, Bu W, Wang X, Ruan J, Shi W, Yu S (2025). Pulmonary fibrosis: from mechanisms to therapies. J Transl Med.

[9] Upagupta C, Shimbori C, Alsilmi R, Kolb M (2018). Matrix abnormalities in pulmonary fibrosis. Eur Respir J.

[10] Mukhatayev Z, Adilbayeva A, Kunz J (2024). CTHRC1: an emerging hallmark of pathogenic fibroblasts in lung fibrosis. Cells.

[11] Younesi FS, Miller AE, Barker TH, Rossi FM, Hinz B (2024). Fibroblast and myofibroblast activation in normal tissue repair and fibrosis. Nat Rev Mol Cell Biol.

[12] Lendahl U, Muhl L, Betsholtz C (2022). Identification, discrimination and heterogeneity of fibroblasts. Nat Commun.

[13] Leask A (2013). Focal Adhesion Kinase: A key mediator of transforming growth factor beta signaling in fibroblasts. Adv Wound Care (New Rochelle).

[14] White ES, Thomas M, Stowasser S, Tetzlaff K (2022). Challenges for clinical drug development in pulmonary fibrosis. Front. Pharmacol.

[15] Bando M, Chiba H, Miyazaki Y, Suda T (2024). Current challenges in the diagnosis and management of idiopathic pulmonary fibrosis in Japan. Respir Investig.

[16] Wang L-H, Baker NE (2015). E proteins and id proteins: helix-loop-helix partners in development and disease. Dev Cell.

[17] Benezra R, Davis RL, Lockshon D, Turner DL, Weintraub H (1990). The protein id: a negative regulator of helix-loop-helix DNA binding proteins. Cell.

[18] Norton JD (2000). ID helix-loop-helix proteins in cell growth, differentiation and tumorigenesis. J. Cell Sci.

[19] Murre C (2005). Helix-loop-helix proteins and lymphocyte development. Nat. Immunol.

[20] Shang S, Yang C, Chen F, Xiang R-s, Zhang H, Dai S-y (2023). ID1 expressing macrophages support cancer cell stemness and limit CD8+ T cell infiltration in colorectal cancer. Nat. Commun.

[21] Roschger C, Cabrele C (2017). The Id-protein family in developmental and cancer-associated pathways. Cell Commun. Signal.

[22] Nair R, Teo WS, Mittal V, Swarbrick A (2014). ID proteins regulate diverse aspects of cancer progression and provide novel therapeutic opportunities. Mol Ther.

[23] Singh S, Sarkar T, Jakubison B, Gadomski S, Spradlin A, Gudmundsson KO (2022). Inhibitor of DNA binding proteins revealed as orchestrators of steady state, stress and malignant hematopoiesis. Front Immunol.

[24] Kondo M, Cubillo E, Tobiume K, Shirakihara T, Fukuda N, Suzuki H (2004). A role for Id in the regulation of TGF-beta-induced epithelial-mesenchymal transdifferentiation. Cell Death Differ.

[25] Love MI, Huber W, Anders S (2014). Moderated estimation of fold change and dispersion for RNA-seq data with DESeq2. Genome Biol.

[26] Yu G, Wang LG, Han Y, He QY (2012). Clusterprofiler: an r package for comparing biological themes among gene clusters. Omics.

[27] Zhu A, Ibrahim JG, Love MI (2019). Heavy-tailed prior distributions for sequence count data: removing the noise and preserving large differences. Bioinformatics.

[28] Badia IMP, Vélez Santiago J, Braunger J, Geiss C, Dimitrov D, Müller-Dott S (2022). decoupleR: ensemble of computational methods to infer biological activities from omics data. Bioinform Adv.

[29] Garcia-Alonso L, Holland CH, Ibrahim MM, Turei D, Saez-Rodriguez J (2019). Benchmark and integration of resources for the estimation of human transcription factor activities. Genome Res.

[30] Tsukui T, Sun KH, Wetter JB, Wilson-Kanamori JR, Hazelwood LA, Henderson NC (2020). Collagen-producing lung cell atlas identifies multiple subsets with distinct localization and relevance to fibrosis. Nat Commun.

[31] Willis BC, Borok Z (2007). TGF-β-induced EMT: mechanisms and implications for fibrotic lung disease. Am J Physiol Lung Cell Mol Physiol.

[32] Zhang H, Li X, Zhang X, Yuan Y, Zhao C, Zhang J (2024). Quantitative ct analysis of idiopathic pulmonary fibrosis and correlation with lung function study. BMC Pulm Med.

[33] Tong Z, Cui Q, Wang J, Zhou Y (2019). Transmir v2. 0: an updated transcription factor-micro rna regulation database. Nucleic Acids Res.

[34] Bhattacharyya S, Wu M, Fang F, Tourtellotte W, Feghali-Bostwick C, Varga J (2011). Early growth response transcription factors: key mediators of fibrosis and novel targets for anti-fibrotic therapy. Matrix Biol.

[35] B Bhattacharyya S, Fang F, Tourtellotte W, Varga J (2013). Egr-1: new conductor for the tissue repair orchestra directs harmony (regeneration) or cacophony (fibrosis). J Pathol.

[36] Alvarado-Vasquez N, Rangel-Escareño C, de Jesús Ramos-Abundis J, Becerril C, Negrete-García MC (2024). The possible role of hypoxia-induced exosomes on the fibroblast metabolism in idiopathic pulmonary fibrosis. Biomed Pharmacother.

[37] Senavirathna LK, Huang C, Yang X, Munteanu MC, Sathiaseelan R, Xu D (2018). Hypoxia induces pulmonary fibroblast proliferation through nfat signaling. Sci Rep.

[38] Ju X, Wang K, Wang C, Zeng C, Wang Y, Yu J (2024). Regulation of myofibroblast dedifferentiation in pulmonary fibrosis. Respir Res.

[39] Forrest S, McNamara C (2004). Id family of transcription factors and vascular lesion formation. Arterioscler Thromb Vasc Biol.

[40] Ruzinova MB, Benezra R (2003). Id proteins in development, cell cycle and cancer. Trends Cell Biol.

[41] Iavarone A, Lasorella A (2006). Id proteins as targets in cancer and tools in neurobiology. Trends Mol Med.

[42] Perk J, Iavarone A, Benezra R (2005). Id family of helix-loop-helix proteins in cancer. Nat Rev Cancer.

[43] Antonângelo L, Tuma T, Fabro A, Acencio M, Terra R, Parra E (2016). Id-1, Id-2, and Id-3 co-expression correlates with prognosis in stage I and II lung adenocarcinoma patients treated with surgery and adjuvant chemotherapy. Exp Biol Med (Maywood).

[44] Poveda-Garavito N, Orozco Castaño CA, Torres-Llanos Y, Cruz-Rodriguez N, Parra-Medina R, Quijano S (2024). Id1 and id3 functions in the modulation of the tumour immune microenvironment in adult patients with B-cell acute lymphoblastic leukaemia. Front. Immunol.

[45] Zhang H, Lawson WE, Polosukhin VV, Pozzi A, Blackwell TS, Litingtung Y (2007). Inhibitor of differentiation 1 promotes endothelial survival in a bleomycin model of lung injury in mice. Am J Pathol.

[46] Lin L, Zhou Z, Zheng L, Alber S, Watkins S, Ray P (2008). Cross talk between Id1 and its interactive protein dril1 mediate fibroblast responses to transforming growth factor-beta in pulmonary fibrosis. Am J Pathol.

[47] Madala SK, Schmidt S, Davidson C, Ikegami M, Wert S, Hardie WD (2012). MEK-ERK pathway modulation ameliorates pulmonary fibrosis associated with epidermal growth factor receptor activation. Am J Respir Cell Mol Biol.

[48] Madala SK, Edukulla R, Phatak M, Schmidt S, Davidson C, Acciani TH (2014). Dual targeting of mek and pi3k pathways attenuates established and progressive pulmonary fibrosis. PloS one.

[49] Blumer S, Fang L, Chen WC, Khan P, Hostettler K, Tamm M (2021). Ipf-fibroblast erk1/2 activity is independent from microrna cluster 17-92 but can be inhibited by treprostinil through dusp1. Cells.

[50] Fortier SM, Penke LR, King D, Pham TX, Ligresti G, Peters-Golden M (2021). Myofibroblast dedifferentiation proceeds via distinct transcriptomic and phenotypic transitions. JCI Insight.

[51] Gupta S, Martin LM, Sinha NR, Smith KE, Sinha PR, Dailey EM (2020). Role of inhibitor of differentiation 3 gene in cellular differentiation of human corneal stromal fibroblasts. Mol Vis.

[52] Chen Z, Shen G, Tan X, Qu L, Zhang C, Ma L (2021). Id1/id3 mediate the contribution of skin fibroblasts to local nerve regeneration through Itga6 in wound repair. Stem Cells Transl Med.

[53] Lowery JW, Frump AL, Anderson L, DiCarlo GE, Jones MT, de Caestecker MP (2010). ID family protein expression and regulation in hypoxic pulmonary hypertension. Am J Physiol Regul Integr Comp Physiol.

[54] Gadomski S, Singh SK, Singh S, Sarkar T, Klarmann KD, Berenschot M (2020). Id1 and Id3 maintain steady-state hematopoiesis by promoting sinusoidal endothelial cell survival and regeneration. Cell Rep.

[55] Pan L, Sato S, Frederick JP, Sun X-H, Zhuang Y (1999). Impaired immune responses and b-cell proliferation in mice lacking the Id3 gene. Mol Cell Biol.

[56] Fraidenraich D, Stillwell E, Romero E, Wilkes D, Manova K, Basson CT (2004). Rescue of cardiac defects in id knockout embryos by injection of embryonic stem cells. Science.

[57] Zhao Q, Beck AJ, Vitale JM, Schneider JS, Gao S, Chang C (2011). Developmental ablation of id1 and id3 genes in the vasculature leads to postnatal cardiac phenotypes. Dev Biol.

[58] Wojnarowicz PM, e Silva RL, Ohnaka M, Lee SB, Chin Y, Kulukian A (2019). A small-molecule pan-id antagonist inhibits pathologic ocular neovascularization. Cell Rep.

[59] Arianti R, Vinnai BÁ, Alrifai R, Karadsheh G, Al-Khafaji YQ, Póliska S (2024). Upregulation of inhibitor of dna binding 1 and 3 is important for efficient thermogenic response in human adipocytes. Sci Rep.

[60] Wojnarowicz PM, Escolano MG, Huang Y-H, Desai B, Chin Y, Shah R (2021). Anti-tumor effects of an id antagonist with no observed acquired resistance. npj Breast Cancer.

[61] Wojnarowicz PM, Lima ESR, Ohnaka M, Lee SB, Chin Y, Kulukian A (2019). A small-molecule pan-id antagonist inhibits pathologic ocular neovascularization. Cell Rep.

