# Supplementary material for: Simultaneous inhibition of ID1 and ID3 mitigates fibroblast activation via cell cycle and MEK/ERK pathways in pulmonary fibrosis
Source: Theranostics. 2026 Apr 16;16(11):6081–98. doi: 10.7150/thno.127118 (PMC13142238; doi:10.7150/thno.127118)
Supplement: Supplementary file 1 — Supplementary figures and tables 1-3; Table S1 provides the characteristics of the human patients; Table S2 lists the primer sequences used for real-time qPCR; Table S3 provides the antibodies used for western blot analysis. [file thnov16p6081s1.pdf]

Supplementary Material

Simultaneous inhibition of ID1 and ID3 mitigates fibroblast activation via cell cycle and MEK/ERK pathways in pulmonary fibrosis

Samar A Antar[1,2], Eric Mensah[1,3], Jacob Dahlka[1], Mark Renton[1], Ahmed A Raslan[4,5,6], Michael Aziz[1], Aymen Halouani[1], Seun Imani[1], Aanandi Parashar[1], Scott Johnstone[1], Robert Benezra[7], Diego Fraidenraich[8], Giovanni Ligresti[4,5] and Yassine Sassi[1,3,9,10]

[1] Fralin Biomedical Research Institute at Virginia Tech Carilion, Virginia, USA

[2] Department of Pharmacology and Biochemistry, Faculty of Pharmacy, Horus University-Egypt, New Damietta 34518, Egypt.

[3]Icahn School of Medicine at Mount Sinai, New York, USA

[4]Arthritis and Autoimmune Diseases Center, Department of Medicine, Boston University Chobanian and Avedisian School of Medicine, Boston, MA, USA

[5]Pulmonary Center, Department of Medicine, Boston University Chobanian and Avedisian School of Medicine, Boston, MA, USA

[6] Department of Zoology, Faculty of Science, Assiut University, Assiut, Egypt

[7] Cancer Biology and Genetics Program, Sloan Kettering Institute, Memorial Sloan Kettering Cancer Center, New York, NY, USA

[8] Department of Cell Biology & Molecular Medicine, Rutgers New Jersey Medical School, Newark, New Jersey, USA

[9] Department of Biomedical Sciences and Pathobiology, Virginia-Maryland College of Veterinary Medicine, Virginia Tech, Blacksburg, Virginia, USA

[10] Department of Internal Medicine, VTC School of Medicine, Roanoke, Virginia, USA

Correspondence to: Yassine Sassi, PhD, Center for Vascular and Heart Research, Fralin Biomedical Research Institute at Virginia Tech Carilion, Roanoke, VA, 24016, Email sassiy@vt.edu.

SUPPLEMENTARY METHODS

BLM mouse model

All the animal experiments were performed in accordance with NIH Guide for the Care and Use of Laboratory Animals. Approval was obtained from Institutional Animal Care at Mount Sinai and Virginia Tech. C57BL/6 male mice were anesthetized with ketamine/xylazine before being immobilized in the supine position on the operating field, and intubated using a 20G catheter. The BLM solution (2 U/kg) was delivered by direct injection into the trachea using a syringe. Once delivery was complete, the needle was withdrawn, and the animals were extubated and placed in a warm environment until they fully recovered from anesthesia, after which they were returned to their respective cages.

ID1/ID3 double Knockout study: Wild-type (WT) and ID1/ID3 knockout (KO) mice (6 weeks old) were used in this study. Mice received daily intraperitoneal injections of tamoxifen (TAM; 40 mg/kg; Sigma) for five consecutive days. Two weeks after completion of tamoxifen treatment, mice were administered a single intratracheal dose of bleomycin (2 U/kg). Four weeks after bleomycin administration, lung function measurements were performed, and lung tissues were harvested for further analysis.

AGX51 study: Mice received a single intratracheal dose of bleomycin (2 U/kg). Two weeks later, AGX51 treatment (50 mg/kg) was initiated. AGX51 was administered via intraperitoneal injection twice daily, three days per week, for two weeks. At the end of the treatment period, lung function measurements were performed, and lung tissues were collected for further analysis.

AAV Study: Mice were administered a single intratracheal dose of bleomycin (2 U/kg). Two weeks later, AAV control or AAV-shID1/ID3 (3×10^{11} genome copies per mouse) was delivered as a single intratracheal dose. Two weeks after AAV administration, lung function measurements were performed, and lung tissues were subsequently collected for further analysis.

Pirfenidone and Nintedanib Comparative Study: Bleomycin was administered as a single intratracheal dose (2 U/kg). Two weeks later, treatment was initiated with AGX51 (50 mg/kg, intraperitoneally, twice daily, three days per week), pirfenidone (100 mg/kg, orally, once daily), or nintedanib (50 mg/kg, orally, once daily). After two weeks of treatment, lung function measurements were performed, and lung tissues were subsequently collected for further analysis.

siRNAs transfection

HLF isolated from healthy donors or from patients with IPF were routinely subcultured to maintain exponential growth. Cells were plated on 12-well plates at 50-70% confluency in complete medium and maintained in a 37°C incubator with 5% CO_2 before being transfected one day later with the different siRNAs. Lipofectamine was used for siRNA transfection following the manufacturer's protocol (ThermoFisher). The final concentrations of siRNA transfected were 5nM for siRNA-ID1(S7104, ThermoFisher), siRNA-ID3 (S7112, ThermoFisher) and negative control siRNA (4390844, ThermoFisher). 24 hrs post-transfection, cells were treated with TGF-β1 (5 ng/mL) for 48 hours and subsequently harvested for RNA and protein extraction. To assess the effect of hypoxia, HLFs were transfected with a HIF-1α specific siRNA (s6539, ThermoFisher) or a control siRNA (4390844, ThermoFisher) at a final concentration of 5nM. Following transfection, cells were incubated either under normoxic conditions (21 %O_2) or under hypoxic conditions (1% O_2) for 48 h. After incubation, total RNA was isolated for subsequent analyses.

Cell proliferation

Fibroblast proliferation was measured by 5-bromo-2'-deoxyuridine (BrdU) incorporation. NHLF and IPF cells were seeded at 4000 cells/well in 96-well plates and maintained in a 37°C incubator with 5% CO_2 for 24 hours to allow cell attachment before treatment. One day later, the cells were transduced with adenoviruses or transfected with siRNAs. BrdU labeling solution was added 48 hours later and incubated for an additional 24 hours to allow incorporation into newly synthesized DNA. Cell proliferation was quantified using the Cell Proliferation ELISA BrdU kit (Sigma-Aldrich) following the manufacturer's instructions. Experiments were performed in triplicate.

Cell differentiation

NHLFs isolated from healthy donors and from patients with IPF were seeded into 6-well plates at approximately 70% confluency in complete culture medium and maintained at 37 °C in a humidified incubator with 5% CO_2. Cells were treated with the indicated experimental conditions,

followed by stimulation with TGF-β (5 ng/ml). 48 hours later, cells were harvested for RNA and protein extraction for subsequent analyses.

Cell migration

Fibroblasts were seeded at a concentration of 50,000 cells/well in a 12 well plate and allowed to incubate until confluency. A scratch was then made in each well using a 200µl pipette tip. The media was then removed and cells were washed with PBS before receiving the different treatments. The scratches of each well were analyzed by optical microscopy 0 and 48 hours following the induced damage. The area between each edge of the scratch was measured using ImageJ software. To calculate the percentage of wound area we used the formula: Wound area (%) =
(Initial Wound Width-Final Wound Width/Initial Wound Width) ×100.

RNA extraction

Total RNA was extracted from cells or homogenized tissues using TRIzol reagent (ThermoFisher) according to the manufacturer's instructions. Briefly, samples were homogenized in TRIzol, and following phase separation, the aqueous phase containing RNA was collected. RNA was precipitated with isopropanol, washed with 75% ethanol, and resuspended in RNase-free water. To remove residual DNA, RNA samples were treated with DNase I (Thermo Scientific) as recommended by the manufacturer. RNA concentration and purity were assessed using a NanoDrop spectrophotometer (Thermo Scientific), and samples were stored at -80 °C until further use.

Reverse transcription

Total RNA (500 ng) was used as a template for complementary DNA (cDNA) synthesis using a High-Capacity cDNA Reverse Transcription Kit (Applied Biosystems) following the manufacturer's instructions. The reaction was performed at 25 °C for 10 min, 37 °C for 120 min, and 85 °C for 5 min, and the cDNA was stored at –20 °C for qPCR analysis.

Tissues harvest

After completion of the lung function measurements, mice were perfused with phosphate-buffered saline (PBS) to remove blood from the pulmonary circulation. The lungs were subsequently

excised and processed for further analyses. Portions of the lung tissue were snap-frozen for RNA and protein extraction. Additional lung samples were embedded in optimal cutting temperature (OCT) compound for histopathological analysis.

Hydroxyproline content

Hydroxyproline was measured using the Hydroxyproline Assay Kit (Sigma-Aldrich, (MAK569-1KT) according to manufacturer's instructions. Left lung lobes were homogenized and 10 mg used for the quantification of the hydroxyproline content that was measured as an indicator of collagen accumulation, using a colorimetric assay. Briefly, approximately 10 mg of lung tissue was homogenized in 100 µL of distilled water and transferred to a pressure-tight polypropylene vial with a PTFE-lined cap. Subsequently, 100 µL of concentrated hydrochloric acid (HCl) was added, and the samples were hydrolyzed at 120 °C for 3 h. After hydrolysis, samples were centrifuged and 10 µL of the supernatant was transferred to a 96-well plate. The samples were then evaporated to dryness in a 60 °C oven. For hydroxyproline detection, dried samples were subjected to oxidation with chloramine-T to generate a reactive intermediate. This intermediate subsequently reacted with *p*-dimethylaminobenzaldehyde (Ehrlich's reagent) to form a chromophore. The absorbance was measured spectrophotometrically at 560 nm after incubation at 60 °C. Hydroxyproline concentrations were calculated by comparison with a hydroxyproline standard curve and expressed relative to tissue weight.

Sirius Red Fast Green staining

Lung tissues were collected, infused with a PBS/OCT mixture (50:50), and stored in OCT at -80°C. Sections (8 µm) were carefully sliced and affixed to Color Frost glass slides (ThermoFisher Scientific). For histological analysis, lung tissue sections were subjected to Sirius Red staining Fast Green FCF (F7252-5G, Sigma-Aldrich), Direct Red 80 (365548-5G, Sigma-Aldrich). Images were obtained using a light microscope. Image J software was used to quantify the collagen deposition. The Ashcroft score was used to assess pulmonary fibrosis using a validated semi-quantitative approach. This scoring system ranges from 0 (normal lung) to 8 (complete fibrous obliteration of the field).

Masson trichrome

Lung tissues embedded in optimal cutting temperature (OCT) compound were cryosectioned at a thickness of 8 μm and mounted onto glass slides. Tissue sections were fixed in 4% paraformaldehyde, rinsed with distilled water, and stained using Masson's Trichrome according to the manufacturer's instructions. Briefly, sections were allowed to cool and then incubated in Bouin's solution for 1 h at 57 °C to enhance staining intensity. Slides were subsequently washed under running tap water to remove the yellow coloration. Sections were then sequentially stained with Weigert's iron hematoxylin to visualize nuclei, followed by Biebrich scarlet-acid fuchsin to stain cytoplasm and muscle fibers. The sections were differentiated in phosphomolybdic-phosphotungstic acid and counterstained with aniline blue to detect collagen fibers. After dehydration through 95% and 100% ethanol and clearing in xylene, the slides were mounted with coverslips. Stained sections and collagen deposition were evaluated using a light microscope.
Fibrosis was assessed using the Ashcroft scoring system, and the investigators were by blinded during analyses.

β-Galactosidase staining

Cellular senescence in lung sections from middle-aged mice was assessed using a Senescence β-galactosidase staining kit (Cell Signaling Technology, #9860) following the manufacturer's instructions. Briefly, fresh-frozen lung sections were brought to room temperature, rinsed with PBS, and fixed with 1× fixative solution for 15 min. After fixation and washing, sections were incubated with freshly prepared β-galactosidase staining solution containing X-gal and maintained at 37 °C in a dry, CO_2-free incubator until blue staining developed. Following incubation, sections were rinsed and visualized using bright-field microscopy. Senescent cells were identified by characteristic blue cytoplasmic staining, and the β-galactosidase-positive area was quantified using ImageJ.

SUPPLEMENTARY FIGURES

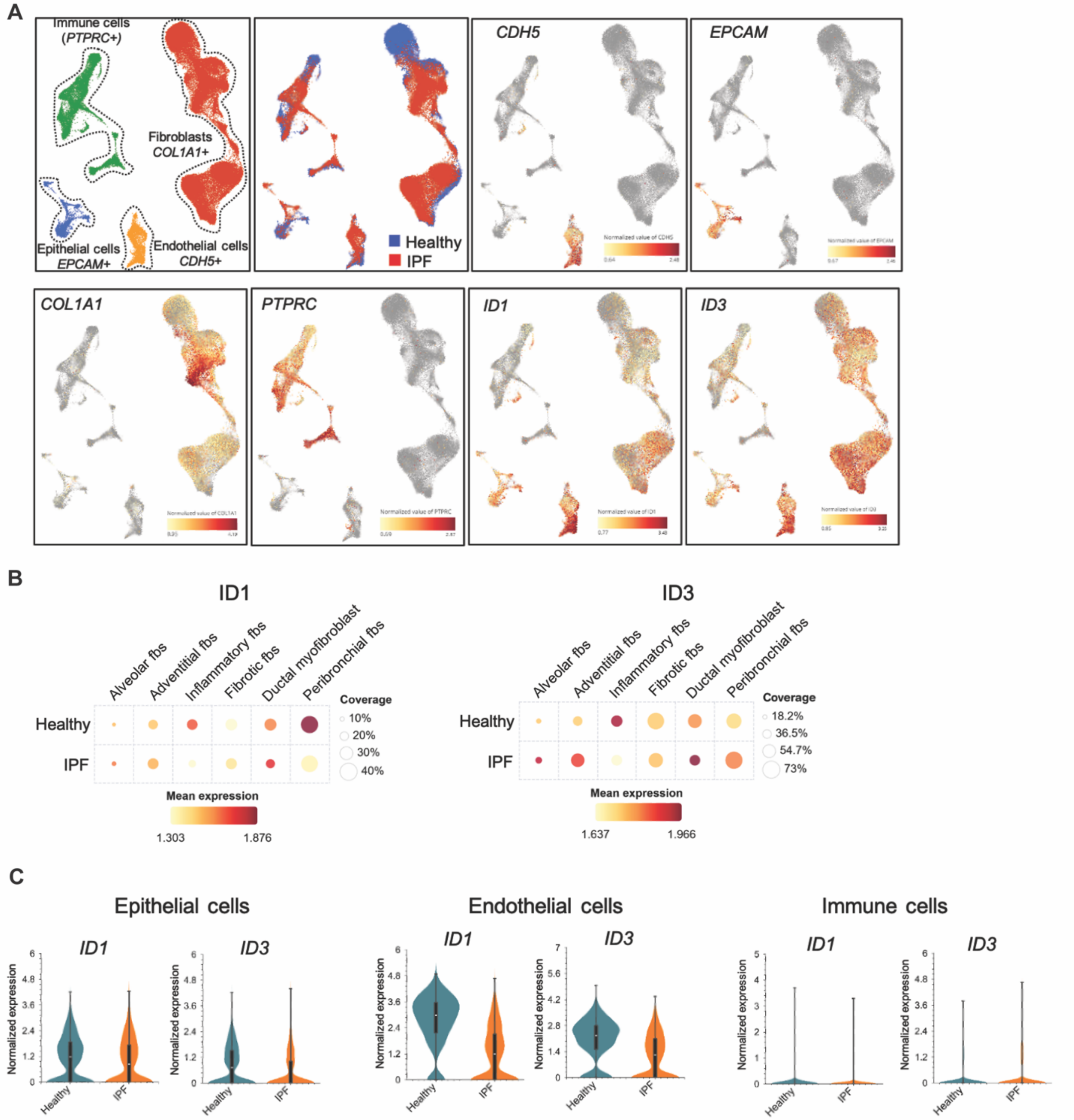

Figure S1. Single-cell RNA sequencing identifies fibroblast-enriched upregulation of ID1 and ID3 in IPF lungs. (A) UMAP plots illustrating the distribution of major lung cell populations, including epithelial, endothelial, fibroblast, and immune cells, in healthy and IPF lungs. The plots display the expression of representative cell-type marker genes: CDH5 (endothelial), EPCAM (epithelial), COL1A1 (fibroblast), and PTPRC (immune), as well as ID1 and ID3 expression across healthy and IPF samples. (B) Dot plot showing ID1 and ID3 expression across distinct fibroblast (COL1A1^{+}PDGFRA^{+}) populations in healthy and IPF human lungs. Fibroblast subsets include alveolar (INMT^{+}ITGA8^{+}COL13A1^{+}), adventitial (PI16^{+}MFAP5^{+}), inflammatory (CCL2^{+}SFRP2^{+}), fibrotic (CTHRC1^{+}), peribronchial (CCL^{+}LGR5^{+}), and ductal myofibroblasts (ASPN^{+}WIF1^{+}FGF18^{+}). Dot size represents the proportion of cells expressing the indicated gene, and color intensity reflects standardized expression levels. (C) Violin plots showing ID1 and ID3 expression levels in epithelial, endothelial, and immune cell populations, comparing healthy and IPF lungs.

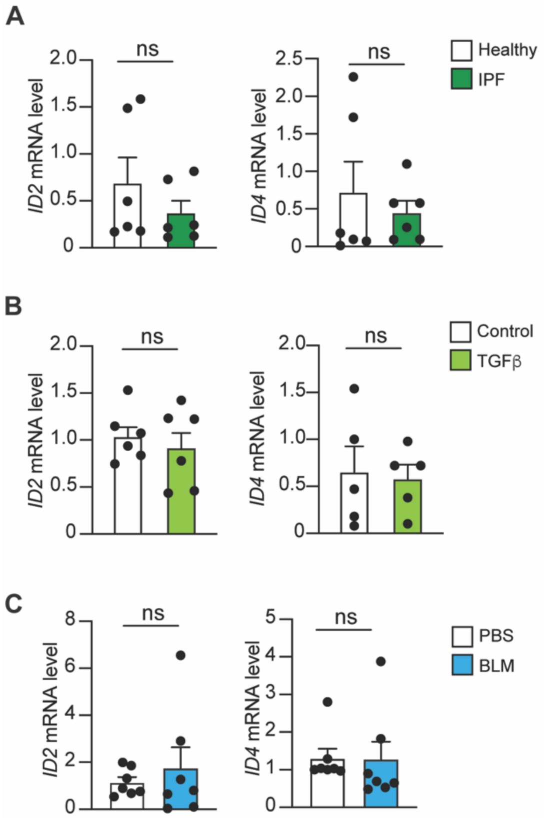

Figure S2. ID2 and ID4 regulation in IPF. (A) ID2 and ID4 mRNA levels determined by real time qPCR analyses in lung fibrobalsts isolated from healthy donors and patients with IPF (n=6/group). ns: not significant by t-test. (B) ID2 and ID4 levels in healthy human lung fibroblasts treated with TGF-β1 (5 ng/ml) for 48 hours. n = 5-6 experiments performed in triplicatens. ns: not significant by t-test. (C) Lung expression of ID2 and ID4 mRNA levels in lung homogenates from control and BLM-treated mice (n = 7 mice/group). ns: not significant by t-test.

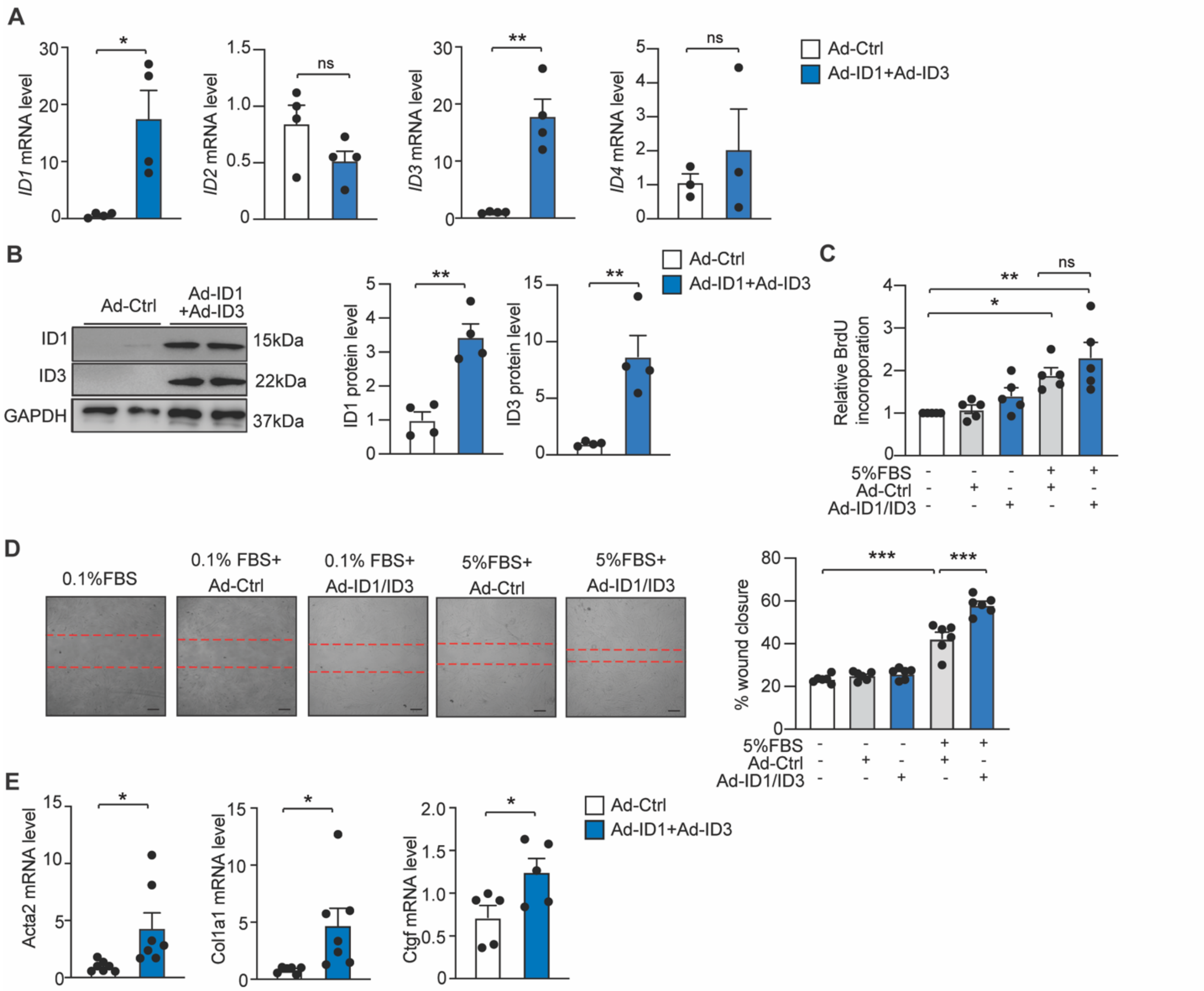

Figure S3. Overexpression of ID1/ID3 increases fibroblast migration and differentiation into myofibroblast. (A) ID1, ID2, ID3 and ID4 mRNA levels in healthy human lung fibroblasts transduced with Ad-Ctrl or Ad-ID1+Ad-ID3 for 48 hours. n =4 experiments performed in duplicate. (B) (Left) Representative immunoblots showing ID1, ID3 and GAPDH protein levels in HLFs transduced with Ad-Ctrl or Ad-ID1+Ad-ID3 for 48 hours. (Right) Quantification of the data. n =4 experiments performed in duplicate. (C) Proliferation of healthy human lung fibroblasts transduced with Ad-Ctrl or Ad-ID1+Ad-ID3 in the presence or absence of serum (5% FBS). n = 5 experiments performed in triplicate. (D) Migration of healthy human lung fibroblasts in the presence of the indicated treatments. n = 6 experiments performed in duplicate. (E) Acta2, Col1a1 and Ctgf mRNA levels in healthy human lung fibroblasts transduced with Ad-Ctrl or Ad-ID1+Ad-ID3 for 48 hours. n = 5-7 experiments performed in duplicate.* $P < 0.05$; ** $P < 0.01$ *** $P < 0.001$ by t-test (A, B, and E) or by one-way ANOVA (C and D).

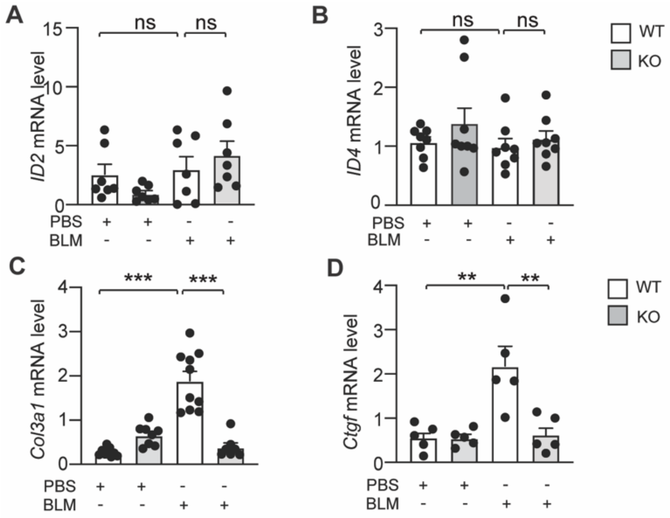

Figure S4. ID1/ID3 deletion decreases Bleomycin-induced lung fibrosis. (A-B) qPCR analysis of ID2 (A) and ID4 (B) mRNA levels in lungs of the indicated groups. n = 7-8 mice/group. (C-D) qPCR analysis of Col3a1 (C) and Ctgf (D) mRNA levels in lungs of the indicated groups. n = 7-10 mice/group in (C) and n= 5 mice/group in (D). ns: not significant, ** $P < 0.01$. *** $P < 0.001$ by two-way ANOVA.

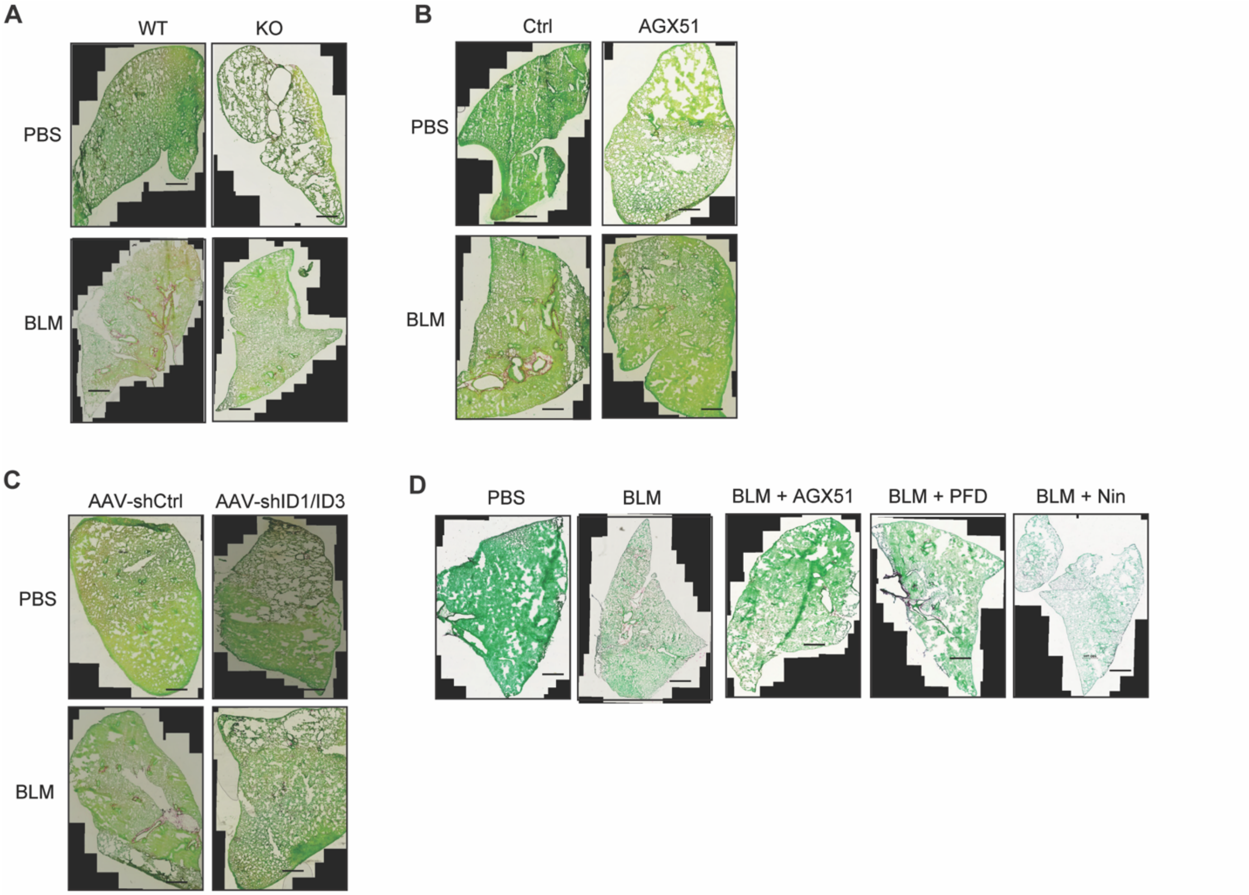

Figure S5. Whole-lung tissue staining with Sirius Red/Fast Green. (A-D) Representative images of Fast Green/Sirius Red-stained lung sections from the indicated groups. Scale bars = 100 μm.

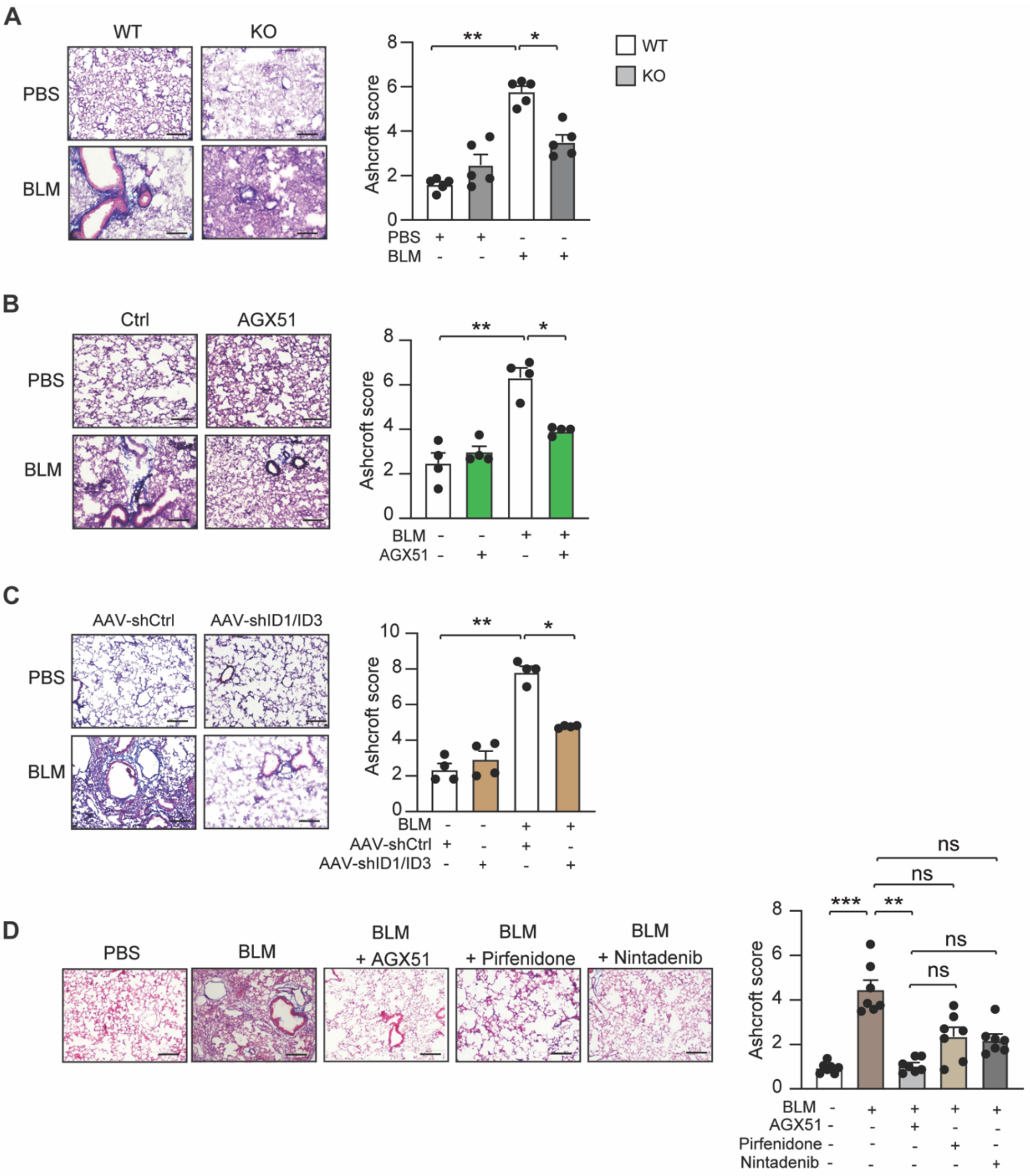

Figure S6: ID1/ID3 inhibition attenuates pulmonary fibrosis in vivo. (A) (Left) Representative images of Masson trichrome stained-lung sections from the indicated groups. Scale bars: 50 μm. (Right) Ashcroft scores representing the extent of fibrosis. n= 5 mice/group. (B) (Left) Representative images of Masson trichrome stained-lung sections from the indicated groups. Scale bars: 50 μm. (Right) Ashcroft scores representing the extent of fibrosis. n= 4 mice/group. (C) (Left) Representative images of Masson trichrome stained-lung sections from the indicated groups. Scale bars: 50 μm. (Right) Ashcroft scores representing the extent of fibrosis. n= 4 mice/group. (D) (Left) Representative images of Masson trichrome stained-lung sections from the indicated groups. Scale bars: 50 μm. (Right) Ashcroft scores representing the extent of fibrosis. n= 7 mice/group. ns: not significant, * $P < 0.05$; ** $P < 0.01$; *** $P < 0.001$ by Kruskal-Wallis followed by Dunn's multiple comparison test.

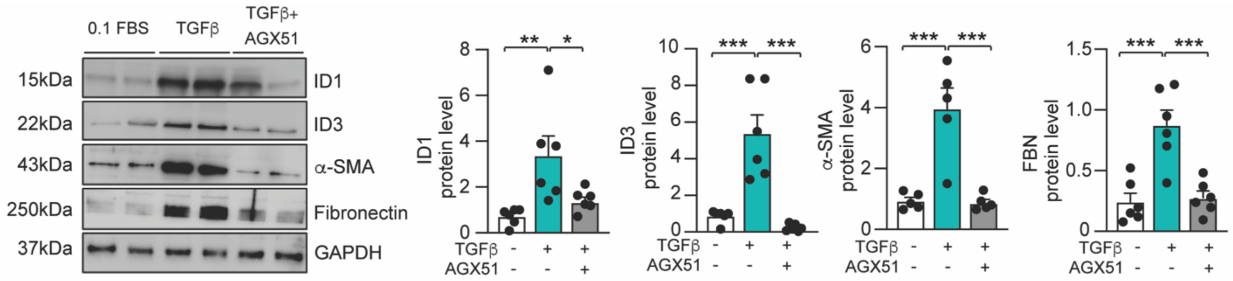

Figure S7. Pharmacological inhibition of ID1/ID3 reduces HLF differentiation. (Left) Representative immunoblots showing ID1, ID3, Acta2, Fibronectin and GAPDH protein levels in human lung fibroblast treated with TGF-β (5 ng/ml) in the presence or absence of AGX51 (20μM). (Right) Quantification of protein expression. n = 5-6 experiments performed in triplicate. * $P < 0.05$; ** $P < 0.01$. *** $P < 0.001$ by one-way ANOVA.

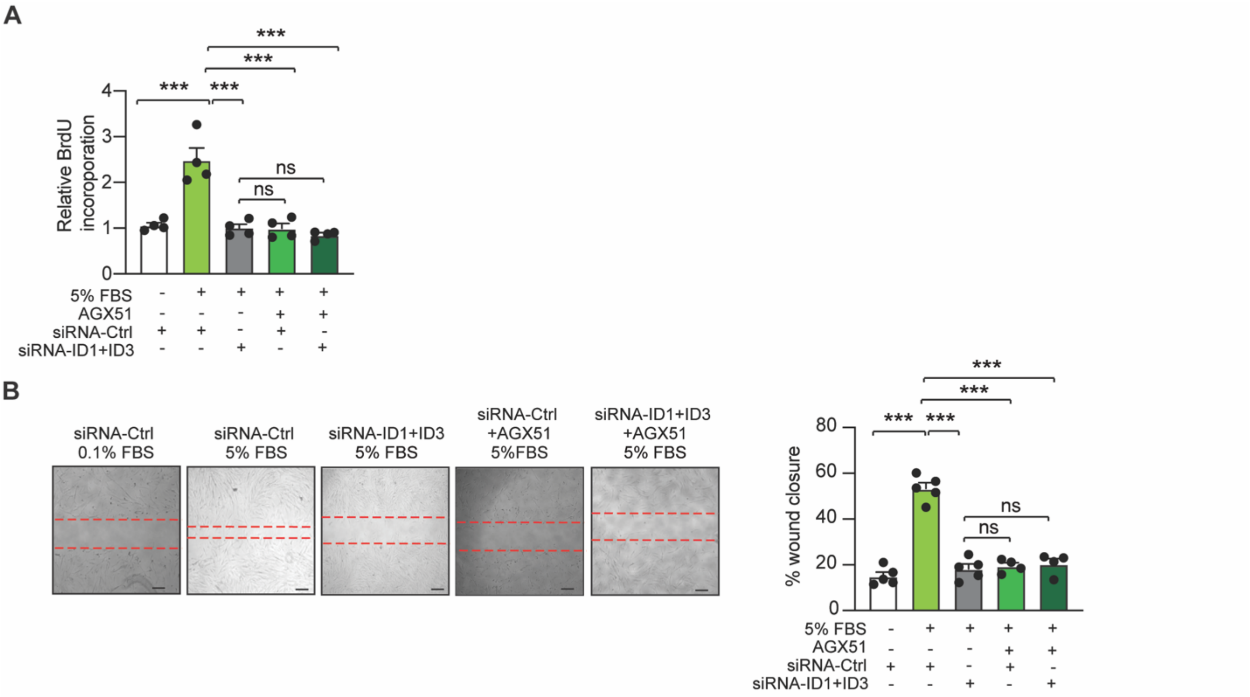

Figure S8. ID1/ID3 silencing recapitulates AGX51-induced phenotypes in HLFs. (A) Proliferation of human lung fibroblasts in the presence of the indicated treatments. n = 4 experiments performed in triplicate. (B) Migration of human lung fibroblasts in the presence of the indicated treatments. n = 5 experiments performed in duplicate. Scale bar: 100 μm. ns: not significant, *** P < 0.001, by one-way-ANOVA.

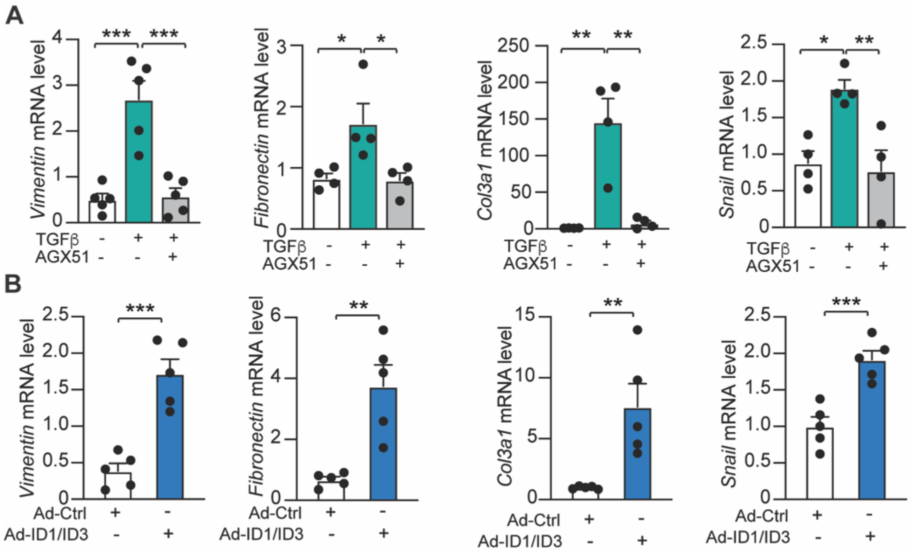

Figure S9. Pharmacological inhibition of ID1/ID3 decreases epithelial-to-mesenchymal transition. (A) Vimentin, Fibronectin, Col3a1, and Snail mRNA levels in human alveolar epithelial cells treated with TGF-β (5 ng/ml) in the presence or absence of AGX51 (20μM) for 48 hours. n =4-5 experiments performed in duplicate. (B) Vimentin, Fibronectin, Col3a1, and Snail mRNA levels levels in human alveolar epithelial cells transduced with Ad-Ctrl or Ad-ID1+Ad-ID3 for 48 hours. n =5 experiments performed in duplicate. * $P < 0.05$; ** $P < 0.01$. *** $P < 0.001$ or one-way ANOVA (A) or by t-test (B).

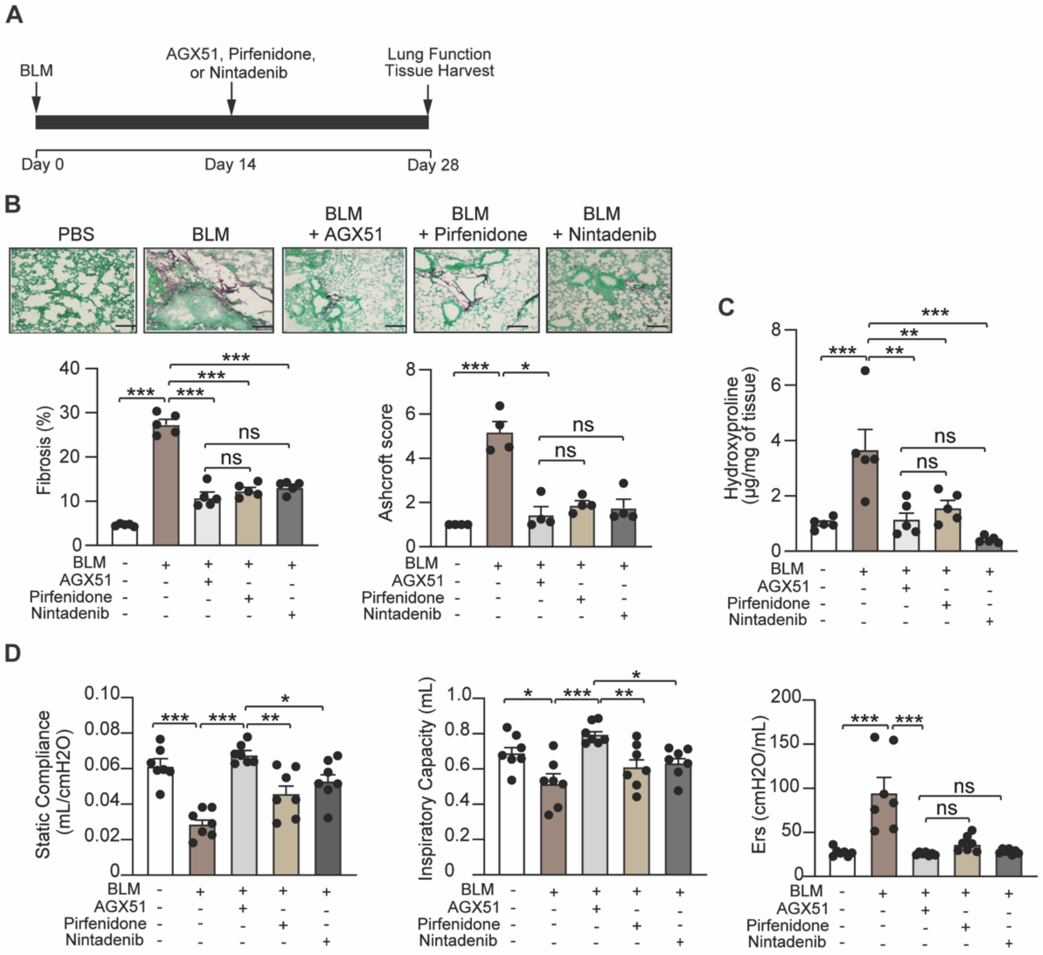

Figure S10. AGX51 may confer greater functional benefit than currently approved antifibrotic therapies. (A) A schematic diagram showing design of the study. (B) Representative images from Fast Green/Sirius-Red-stained lungs of the indicated groups, quantitative analysis of fibrosis, and Ashcroft scores representing the extent of fibrosis. Scale bar: 200 μ m. n = 4-5 mice/group. (C) Hydroxyproline content in lungs from the indicated groups n=5 mice/group. (D) Lung function data parameters for inspiratory capacity, compliance, and single frequency elastance. n=7 mice/group. ns: not significant, * P < 0.05; ** P < 0.01. *** P < 0.001 by one-way ANOVA. Statistical differences in Ashcroft score were analyzed by Kruskal-Wallis test followed by Dunn's multiple-comparison test.

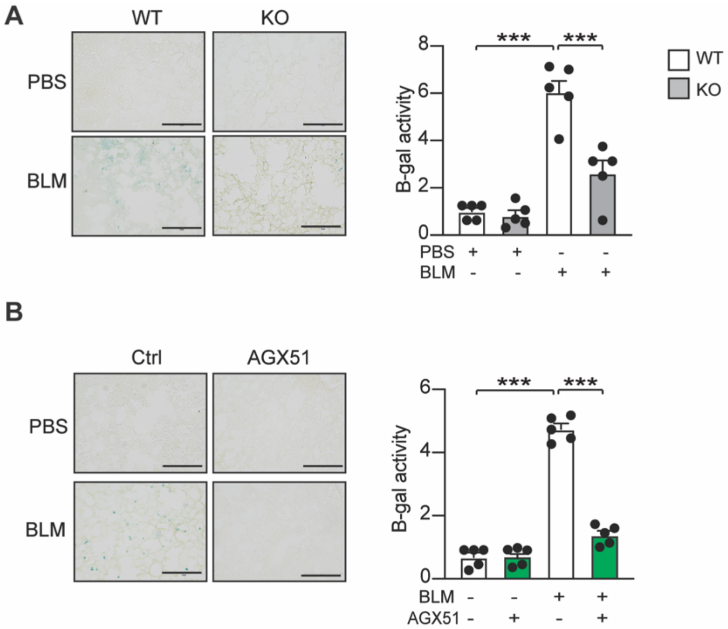

Figure S11. Sensceence-associated-β-galactosidase (SA-β-gal) staining in the lung. (A) (Left) Representative images from SA-β-gal-stained lung sections of the indicated groups. Scale bar: 50 μm. (Right) Quantitative analysis of SA-β-gal staining. n=5 mice/group. (B) (Left) Representative images from SA-β-gal-stained lung sections of the indicated groups. Scale bar: 50 μm. (Right) Quantitative analysis of SA-β-gal staining. n=5 mice/group. *** $P < 0.001$ by two-way ANOVA.

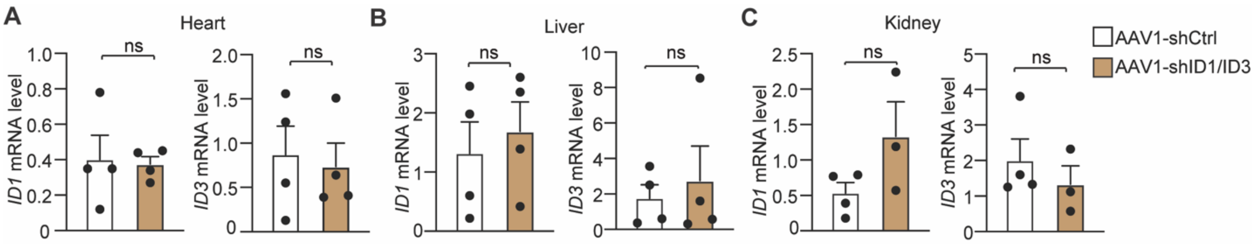

Figure S12. Lung-specific manipulation of ID1 and ID3. PCR analysis of ID1 and ID3 mRNA levels in (A) heart, (B) liver, and (C) kidney of the indicated groups. n = 3-4 mice/group. ns: not significant by t-test.

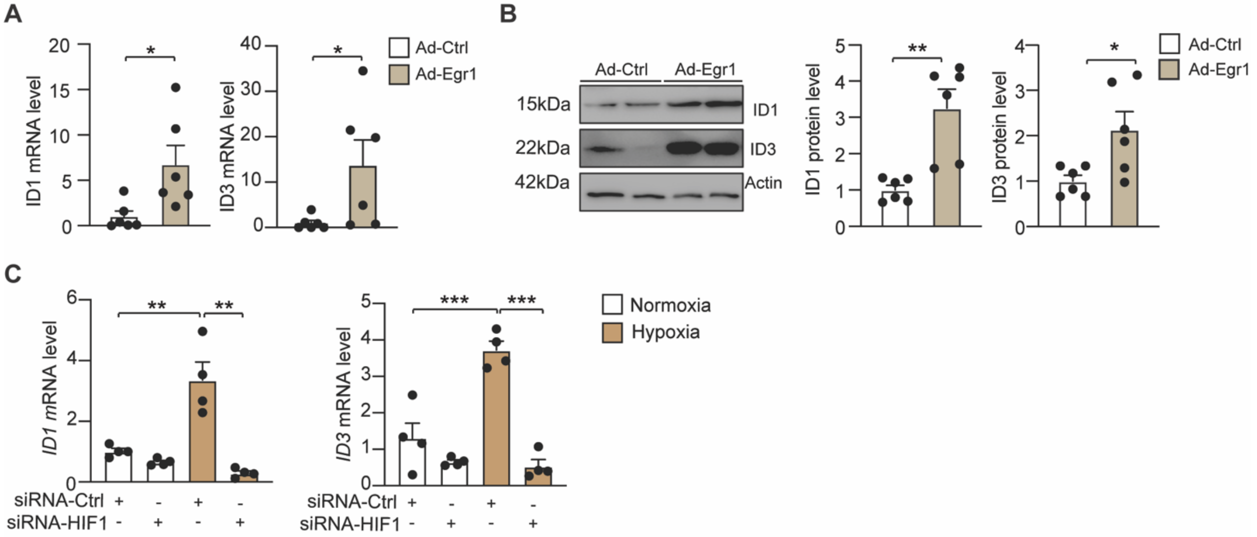

Figure S13: ID1/ID3 regulation by Egr-1 and hypoxia. (A) ID1 and ID3 mRNA levels in human lung fibroblasts transduced with Ad-Ctrl or Ad-Egr-1. n = 6 experiments performed in duplicate. (B) ID1 and ID3 protein levels in C2C12 cells transduced with Ad-Ctrl or Ad-Egr-1. n = 6 experiments. (C) ID1 and ID3 mRNA levels in human lung fibroblasts exposed to normoxic (21% O_2) or hypoxic (1% O_2) conditions for 48 hours in cells transfected with siRNA-HIF1 or siRNA-Ctrl. n = 4 experiments performed in duplicate. * $P < 0.05$; ** $P < 0.01$, *** $P < 0.001$ by t-test (A and B) or 2-way ANOVA (C).

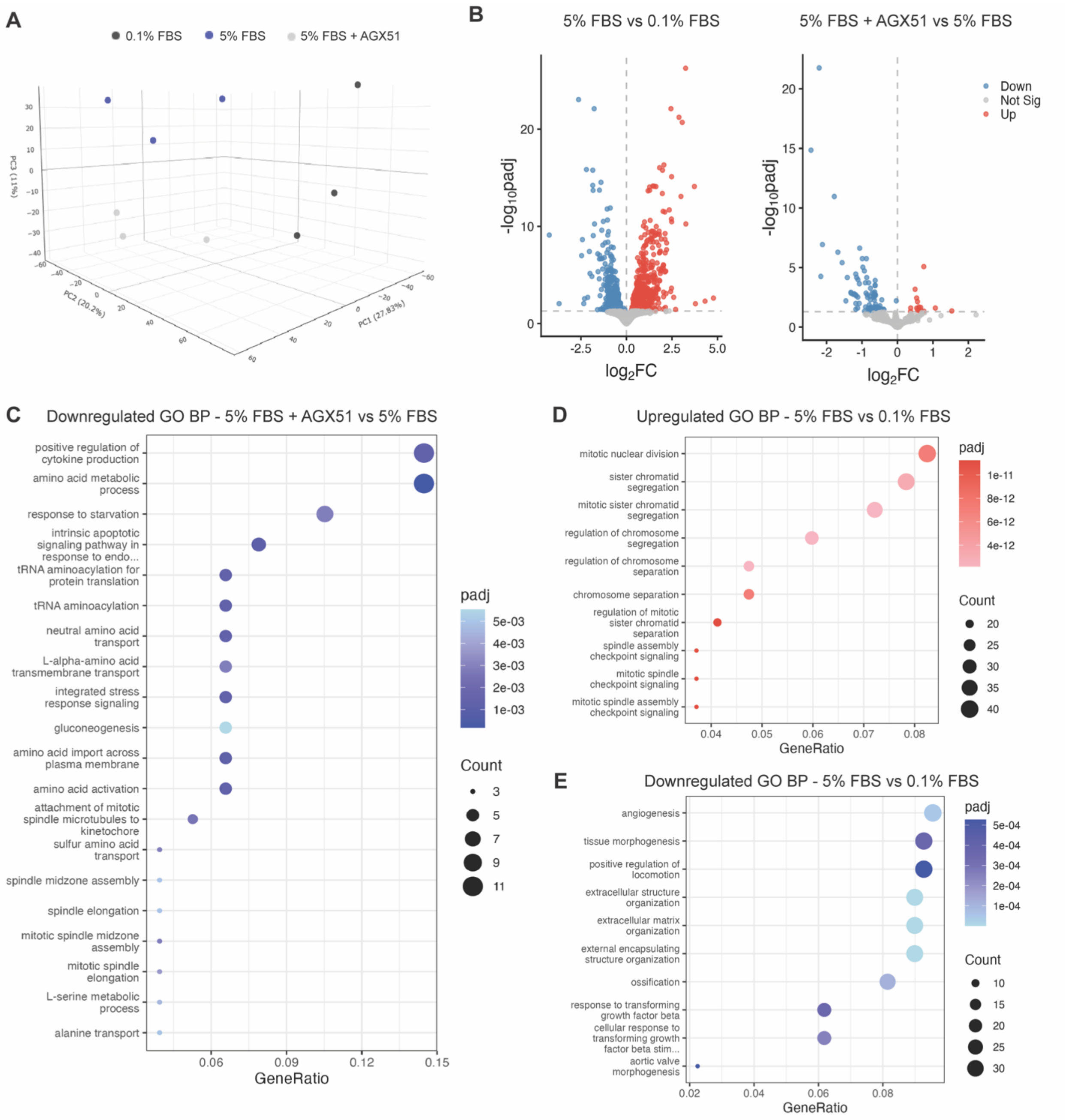

Figure S14. Bulk RNA sequencing of AGX51-treated HLFs. (A) Three-dimensional principal component analysis (PCA) plot derived from DESeq2 normalized and $\log_2$ transformed bulk RNA sequencing counts. (B) Volcano plots of differentially expressed genes (DEGs) identified with |log2(Fold Change (FC))| > 0 and a Benjamini-Hochberg multiple comparison adjusted P value (padj) < 0.05 for indicated group comparisons. (C) Bulk RNA sequencing Gene Ontology (GO) Biological Process (BP) pathway analysis - 5% FBS + AGX51 vs 5% FBS. Top 20 significantly downregulated pathways by Benjamini-Hochberg multiple comparison adjusted P value (padj) following AGX51 treatement in 5% FBS treated cells. No significantly upregulated pathways were detected. (D) Top 10 significantly upregulated pathways by Benjamini-Hochberg multiple comparison adjusted P value (padj) from GO BP pathway analysis following serum treatement (0.1% vs 5% FBS). (E) Top 10 significantly downregulated pathways by Benjamini-Hochberg multiple comparison adjusted P value (padj) from GO BP pathway analysis following serum treatement (0.1% vs 5% FBS).

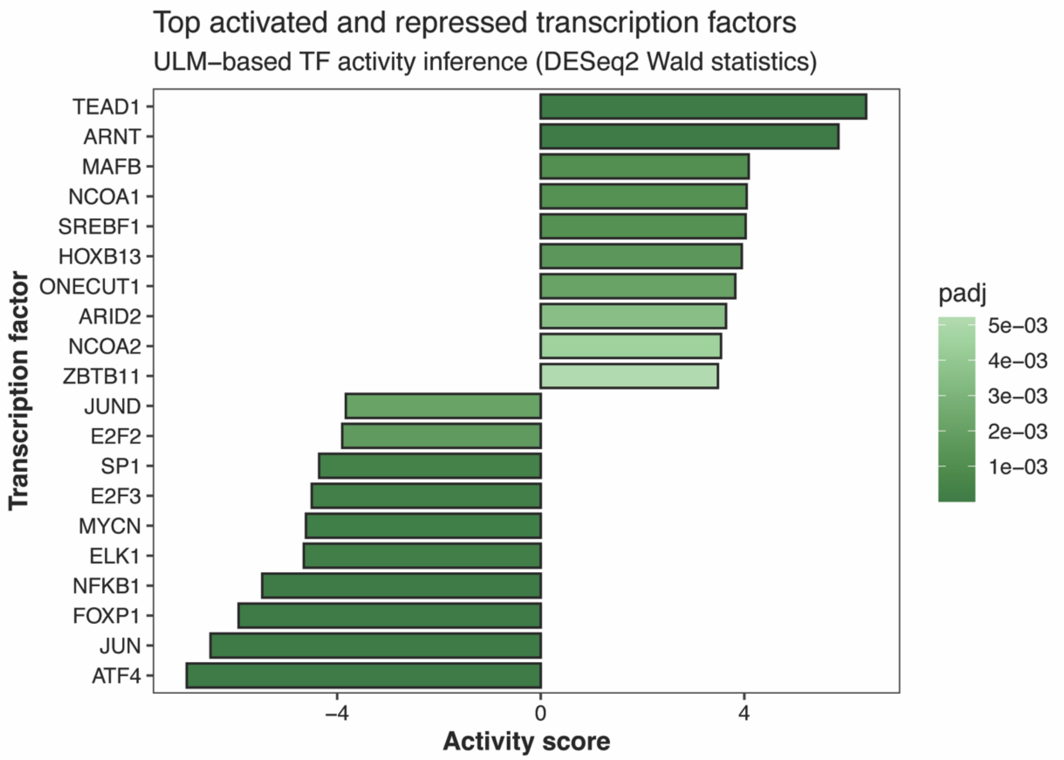

Figure S15. Predicted transcription factors regulated by ID1/ID3 inhibition.TF inference estimated from bulk RNA sequencing differential gene expression using univariate linear model (ULM) with DESeq2 Wald statistic as input. TFs with a positive activity score are predicted to have increased transcriptional activity in response to AGX51 treatment in 5% FBS treated cells. TFs with a negative score are predicted to have decreased transcriptional activity. The top 10 and bottom 10 statistically significant (Bejamini-Hochberg adjusted padj < 0.05) TFs when sorted by activity score are displayed.

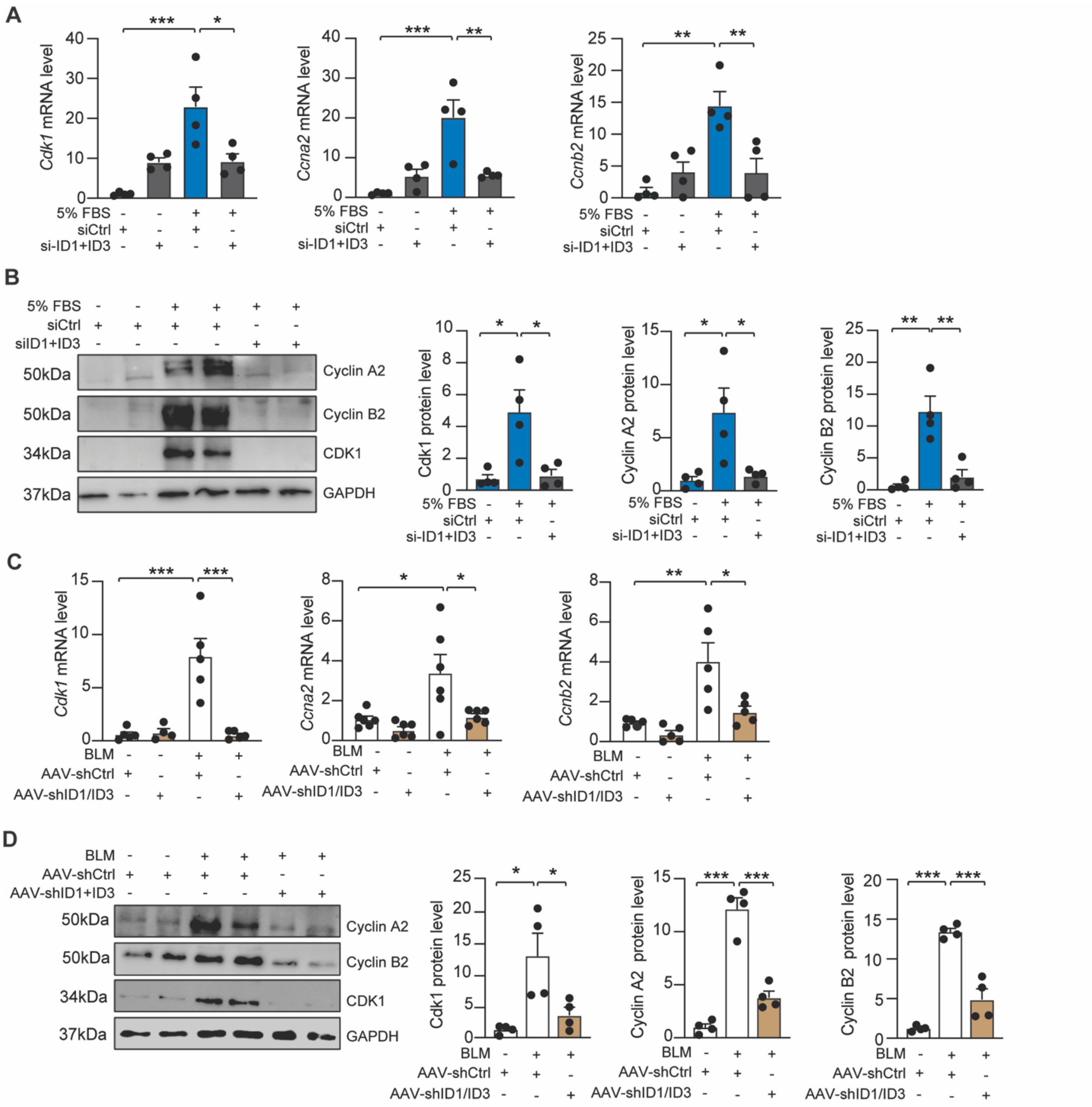

Figure S16: ID1/ID3 exert their effects through modulation of cell cycle-associated genes and activation of the MEK signaling pathway. (A) PCR analysis of Ccna2, Ccnb2 and Cdk1 mRNA levels in HLF treated with siRNA-Ctrl or siRNA-ID1/ID3 n = 4 per group. (B) (Left) Representative immunoblots showing Cyclin A2, Cyclin B2 and Cdk1 protein levels in HLF treated treated with siRNA-Ctrl or siRNA-ID1/ID3. n = 4 per group. (Right) Quantification of the data. (C) Pulmonary Ccna2, Ccnb2 and Cdk1 mRNA levels in lungs from the indicated groups n = 5-6 per group. (D) (Left) Representative immunoblots showing Cyclin A2, Cyclin B2 and Cdk1 protein levels in lungs from the indicated groups. (Right) Quantification of the data. n = 4 per group. * P < 0.05; ** P < 0.01. *** P < 0.001, by one-way ANOVA (B and D) or Two-way ANOVA (A and D).

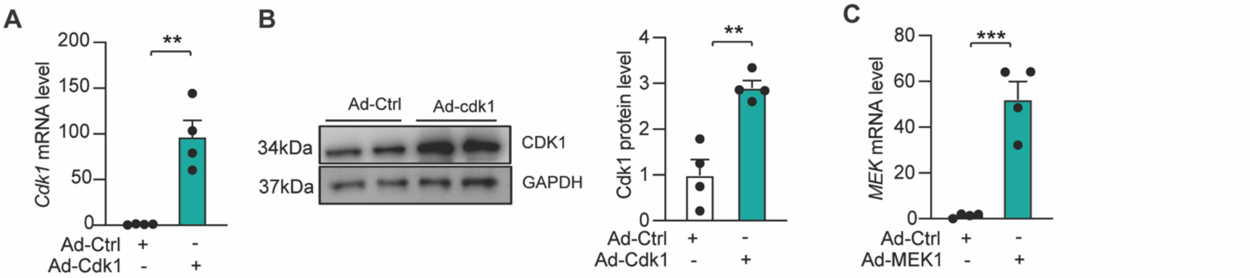

Figure S17: ID1/ID3 exert their effects through modulation of cell cycle-associated genes and activation of the MEK signaling pathway. (A) Cdk1 mRNA levels in HLFs transduced with Ad-Cdk1 or Ad-Ctrl. n = 4 experiments performed in triplicate. (B) (Left) Representative immunoblots showing Cdk1 and GAPDH protein levels in HLFs transduced with Ad-Cdk1 or Ad-Ctrl. (Right) Quantification of the data. n = 4 experiments performed in duplicate. (C) qPCR assessment of MEK1 mRNA levels in HLFs transduced with Ad-MEK1 or Ad-Ctrl. n = 4 experiments performed in triplicate. ** P < 0.01. *** P < 0.001 by t-test.

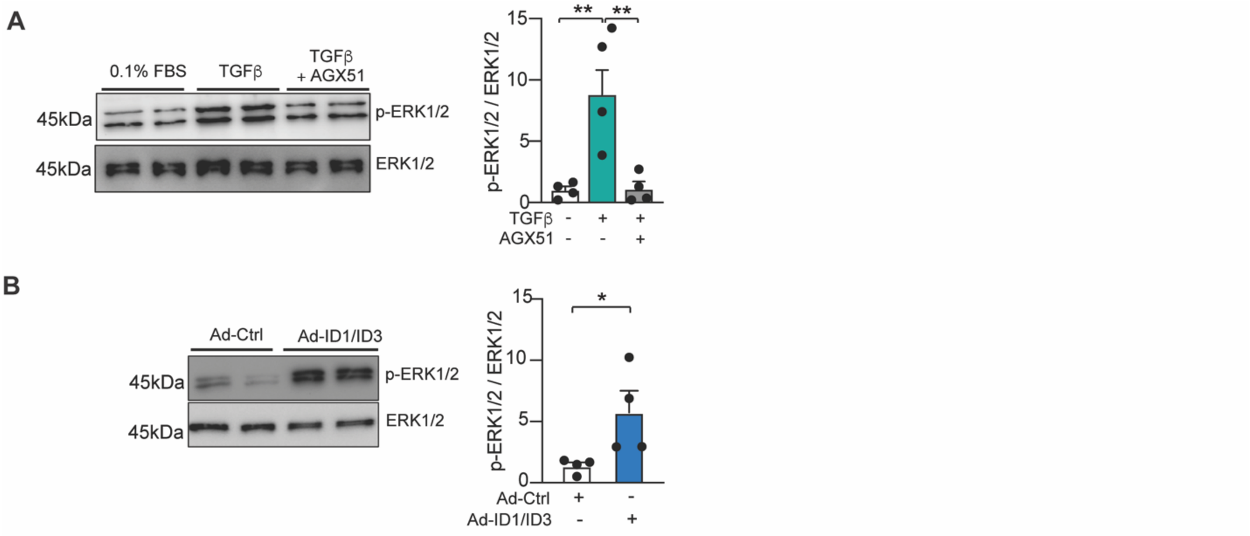

Figure S18. ID1/ID3 regulate the MEK/ERK pathway. (A) (Left) Representative immunoblots showing phosphorylated ERK1/2 and total ERK1/2 protein levels in HLFs treated with TGF-β1 (5 ng/ml) in the absence or presence of AGX51 (20 μM). (Right) Quantification of the data. n = 4. (B) (Left) Representative immunoblots showing phosphorylated ERK1/2 and total ERK1/2 protein levels in HLFs transduced with Ad-Ctrl or Ad-ID1/ID3 for 48 hours. (Right) Quantification of the data. n = 4.* $P < 0.05$; ** $P < 0.01$ by one way ANOVA (A) or by t-test (B).

SUPPELMENTARY TABLES

Table S1. Detailed characteristics of human lung samples used in this study.

	Sex	Age	IPF
**Control 1**	Male	27	-
**Control 2**	Female	20	-
**Control 3**	Male	59	-
**Control 4**	Male	7	-
**Control 5**	Male	42	-
**Control 6**	Male	73	-
**IPF 1**	Female	49	IPF
**IPF 2**	Female	67	IPF
**IPF 3**	Male	62	IPF
**IPF 4**	Female	59	IPF
**IPF 5**	Male	55	IPF
**IPF 6**	Unknown	Unknown	IPF

Table S2. Real-time PCR Primers

Gene	Direction	Primer sequence (5'-3')
Human α-SMA	Forward	ACCCACAATGTCCCCATCTA
	Reverse	GAAGGAATAGCCACGGCTCAG
Human Ccna2	Forward	TCCCCAGACTTTTTCGCTCT
	Reverse	GGATGCCAGTCTTACTCATAGC
Human Ccnb2	Forward	TACTGCTCTGCTCTTGGCTT
	Reverse	GCTGTTCAACATCAACCTCCC
Human Cdk1	Forward	GCCCTTTAGCGCGGATCTAC
	Reverse	AGGAACCCCTTCCTCTTCACT
Human Col1a1	Forward	GGCTCCTGCTCCTCTTAGC
	Reverse	TCTCTTACGCAGGTGATTGGT
Human Col3a1	Forward	GAGATGTCTGGAAGCCAGAACCATG
	Reverse	ATCTCCCTTGGGGCCTTGAGGT
Human Ctgf	Forward	TCCCAAAATCTCCAAGCCTA
	Reverse	GTAATGGCAGGCACAGGTCT
Human Fibronectin	Forward	TCCGGGACTCAATCCAAATG
	Reverse	CCAGGAACCCTGAACTGTAAG
Human/ Mouse Gapdh	Forward	CCAAGGTCATCCATGACAACTT
	Reverse	GTCTTCTGGGTGGCAGTGATG
Human ID1	Forward	CGCATCTTGTGTCGCTGAAG
	Reverse	CCGATCGGTCTTGTTCTCCC
Human ID2	Forward	CCCACTATTGTCAGCCTGCA
	Reverse	CTGCAAGGACAGGATGCTGA
Human ID3	Forward	TACAGCGCGTCATCGACTAC
	Reverse	TGACAAGTTCCGGAGTGA
Human ID4	Forward	TGAGTAGTACCGGGGAGTGGG
	Reverse	AAGAAAAGTAGCCCACCCGG
Human MAP2K1	Forward	TGAAGCTGGAGAGGACCAAC
	Reverse	CTCCCACCTTCTGCTTCTGG

Human Snail1	Forward	GACCCACACTGGCGAGAAG
	Reverse	CATTCGGGAGAAGGTCCGAG
Human Vimentin	Forward	AGGCGAGGAGAGCAGGATTT
	Reverse	AGTGGGTATCAACCAGAGGGA
Mouse Ccna2	Forward	CTCACTACATAGCTGACTTGGAC
	Reverse	CTGGGTGGCGCCTTTAATC
Mouse Ccnb2	Reverse	GTTCTGAGGTTTCTTCGCCA
	Forward	ATCCGGCGGGCAGTTTTAG
Mouse Cdk1	Forward	CTCCTGGGCAGTTCATGGAT
	Reverse	CCACAGCGTCACTACCTCG
Mouse Col1a1	Forward	CTGGCAAGAAGGGAGATGA
	Reverse	CACCATCCAAACCACTGAAA
Mouse Col3a1	Forward	ACAGCAAATTCACTTACACAGTTC
	Reverse	CTCATTGCCTTGCGTGTTT
Mouse Ctgf	Forward	AGAACTGTGTACGGAGCGTG
	Reverse	CCATCTTTGGCAGTGCACAC
Mouse Fibronectin	Forward	ATGAGAAGCCTGGATCCCCT
	Reverse:	CAGTTGGGGAAGCTCATCTGT
Mouse ID1	Forward	ATCATGAAGGTCGCCAGTGG
	Reverse	CCGACAGACCAAGTACCACC
Mouse ID2	Forward	ATCCTGTCCTTGCAGGCATCT
	Reverse	GTCCATTCAACGTGTTCTCCTG
Mouse ID3	Forward	TTTCCGAGAATGGGGTGTCG
	Reverse	ATCAGGGCAGCAGAGCTTTT
Mouse ID4	Forward	GCAGGGTGACAGCATTCTCT
	Reverse	AATTTCTCCTCTGGCCCTCC

Table S3. List of antibodies used.

Antibody	Company	Product number
Anti-mouse IgG, HRP-linked Antibody	Cell Signaling	7076S
Anti-rabbit IgG, HRP-linked Antibody	Cell Signaling	7074S
Anti-Cyclin A2 antibody	Abcam	EPR17351
Beta Actin	Santa Cruz	sc-47778
Cdc2 (E1Z6R) Rabbit mAb	Cell Signaling	28439
Collagen 3 Antibody (B-10)	Santa Cruz Biotechnology	sc-271249
Collagen 1 Antibody (3G3)	Santa Cruz Biotechnology	sc-293182
Cyclin B2 Monoclonal Antibody	ThermoFisher	MA1-156
Fibronectin polyclonal antibody	ProteinTech	15613-1-AP
GAPDH Monoclonal antibody	Proteintech	60004-1-Ig
ID1 Antibody (F-10)	Santa Cruz Biotechnology	sc-365654
ID3 monoclonal antibody (2B11)	ThermoFisher	MA1-23242
MEK1/2 (D1A5) Rabbit mAb	Cell Signaling	8727T
Monoclonal Anti-Actin, α-Smooth Muscle clone 1A4	Sigma Aldrich	A2547
P44/42 MAPK (ERK1/2) (137F5) Rabbit Monoclonal Antibody	Cell Signaling	CST 4695
P-p44/42MAPK (ERK1/2, Thr202/Tyr204) Rabbit Monoclonal Antibody	Cell Signaling	CST 4370
Phospho-MEK1/2 (Ser217/221)	Cell Signaling	9154T
Rabbit Monoclonal Anti-Mouse/Human Id1	Biocheck	BCH-1/#195-14
Rabbit Monoclonal Anti-Mouse/Human Id3	Biocheck	BCH-4/#6-1
